# Deep Genetic Divergence between Disjunct Refugia in the Arctic-Alpine King’s Crown, *Rhodiola integrifolia* (Crassulaceae)

**DOI:** 10.1371/journal.pone.0079451

**Published:** 2013-11-01

**Authors:** Eric G. DeChaine, Brenna R. Forester, Hanno Schaefer, Charles C. Davis

**Affiliations:** 1 Department of Biology, Western Washington University, Bellingham, Washington, United States of America; 2 Department of Environmental Sciences, Western Washington University, Bellingham, Washington, United States of America; 3 Department of Organismal and Evolutionary Biology, Harvard University, Cambridge, Massachusetts, United States of America; CNRS / Université Joseph-Fourier, France

## Abstract

Despite the strength of climatic variability at high latitudes and upper elevations, we still do not fully understand how plants in North America that are distributed between Arctic and alpine areas responded to the environmental changes of the Quaternary. To address this question, we set out to resolve the evolutionary history of the King’s Crown, *Rhodiola integrifolia* using multi-locus population genetic and phylogenetic analyses in combination with ecological niche modeling. Our population genetic analyses of multiple anonymous nuclear loci revealed two major clades within *R. integrifolia* that diverged from each other ~ 700 kya: one occurring in Beringia to the north (including members of subspecies *leedyi* and part of subspecies *integrifolia*), and the other restricted to the Southern Rocky Mountain refugium in the south (including individuals of subspecies *neomexicana* and part of subspecies *integrifolia*). Ecological niche models corroborate our hypothesized locations of refugial areas inferred from our phylogeographic analyses and revealed some environmental differences between the regions inhabited by its two subclades. Our study underscores the role of geographic isolation in promoting genetic divergence and the evolution of endemic subspecies in *R. integrifolia*. Furthermore, our phylogenetic analyses of the plastid spacer region *trn*L-F demonstrate that among the native North American species, *R. integrifolia* and *R. rhodantha* are more closely related to one another than either is to *R. rosea*. An understanding of these historic processes lies at the heart of making informed management decisions regarding this and other Arctic-alpine species of concern in this increasingly threatened biome.

## Introduction

The Quaternary Period (~2.5 Myr–present) has been characterized by rapid oscillations between cold, glacial periods and warm interglacials that have profoundly influenced the distribution and diversity of regional flora and fauna [1,2]. Though the relative strength of climatic variability was arguably strongest at high latitudes and upper elevations, we still do not fully understand how North American arctic-alpine plants responded to these environmental changes. The few high elevation and high latitude North American plants that have been investigated show genetic evidence of isolation by refugia [3,4,5,6], raising biogeographic questions concerning the role of refugia in species' evolution. Furthermore, it remains unclear how differences in environmental conditions across a species’ range have impacted refugia-based lineage divergence. Filling in these gaps will help create a baseline for future monitoring that will be particularly important for the conservation of arctic-alpine plants.

The landscape of North America is topographically heterogeneous, which helped set the stage for climate associated genetic divergence of arctic-alpine plants during the Quaternary. The north-south running mountain chains provided opportunities for the persistence of arctic-alpine plants during cold and warm climatic periods, by maintaining suitable habitat in refugia and permitting the latitudinal migration of taxa in response to cooling and warming climates [7]. The massive sheets of ice that grew over much of the continent, in conjunction with the down-slope expansion of mountain glaciers during cold periods, served to isolate regions and their inhabitants from one another. Several ice-free refugia for tundra species have been identified across the continent, including Beringia north of the ice sheets (encompassing eastern Siberia and far northwestern North America), and parts of the North Cascade Range, the Olympic Peninsula, Haida Gwaii (formerly the Queen Charlotte Islands), the Sierra Nevada Range, and the central and southern Rocky Mountains to the south [8,9,10,11,12]. The cold climate associated with glacial advances also opened opportunities for east-west migration along the tundra-like habitat that skirted the southern edge of the Laurentide Ice Sheet, setting up a glacial refugium in the Driftless Area of the present-day midwestern United States (including southwestern Wisconsin, southern Minnesota, and northeastern Iowa [13]). During warm interglacials, populations would have been restricted to the smaller, remaining patches of tundra habitat at higher elevations, scattered along a chain of sky islands in the western cordillera. Thus, though warming permitted the colonization of deglaciated habitat at new latitudes, such as that of British Columbia and Alberta in Canada, alpine plant populations across the range would have been smaller and less connected during interglacials, with a higher chance of local extinction. By promoting range shifts, but limiting taxa to a patchy distribution scattered across this heterogeneous environment, the Quaternary profoundly influenced the evolutionary history of arctic-alpine plants in North America. 

**Figure 1:**
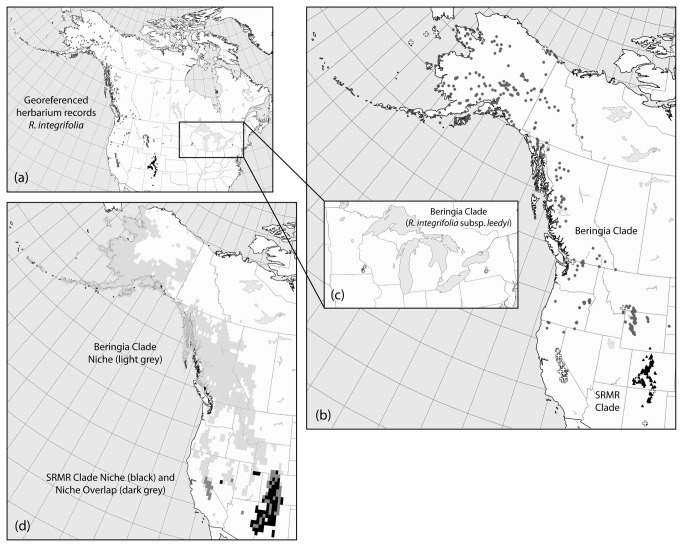
Distribution maps and clade-specific niche models. (a) Distribution of georeferenced herbarium records for *Rhodiola integrifolia* in North America; grey circles represent the Beringia Clade, black triangles represent the Southern Rocky Mountain Refugium (SRMR) clade.  (b) Distribution of herbarium records and specimen data (white crosses) in western North America.  (c) Distribution of herbarium records and specimen data for relict populations in eastern North America, *R. integrifolia* subsp. *leedyi*.  (d) Ensemble ecological niche models based on current climate variables for the Beringia (light grey) and SRMR (black) clades. Overlap in the niche predictions is indicated in dark grey.

Studies focused on plant species that inhabit both arctic and alpine environments hold important clues to understanding the larger history of this region. The King’s Crown, *Rhodiola integrifolia* Raf. (Crassulaceae), is an excellent taxon for investigating the Quaternary history of arctic-alpine North America because of its widespread distribution across the tundra landscape, and its more narrowly distributed subspecies [14]. Like many arctic-alpine taxa in North America, the taxonomy of *R. integrifolia* is debated; there are a number of described subspecies, and the closest relatives in the genus *Rhodiola* are unknown. Ongoing taxonomic uncertainty in this species is due in part to its highly disjunct distribution – with an arctic range from central Russia across the Bering Sea to Alaska, extending southwards along the cordillera to the Cascades, Sierra Nevada, and the Rocky Mountains, with greatly disjunct populations in Minnesota and New York (Figure 1a-c). Given the broad geographic distribution of this infraspecific variation, Quaternary climate and regional topography likely played a major role in diversification within the species [15], making it an ideal candidate for helping to understand the history of this biome. In addition, several of the populations/subspecies of *R. integrifolia* are small and of conservation concern. Thus, clarifying their evolutionary relationships will aid in conservation efforts and potentially identify overlooked, genetically distinct taxa. A better understanding of how *R. integrifolia* responded to environmental changes informs us about the role of the Quaternary in the process of speciation, the generation of endemics in arctic-alpine taxa, and regions important for conservation. 

**Figure 2:**
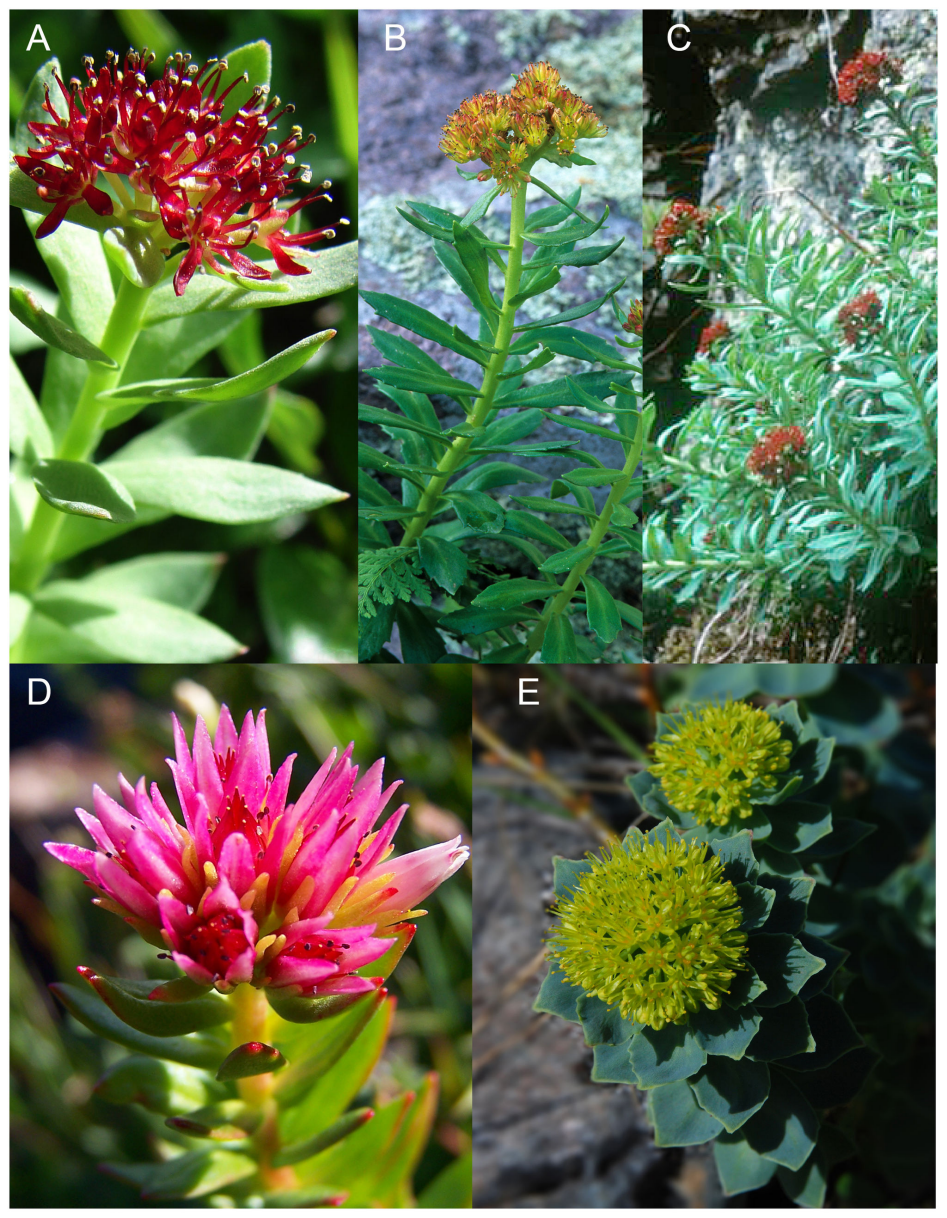
The North American *Rhodiola*. Photo credits are given in parentheses. a) *R. integrifolia* subsp. *integrifolia* (Alfred Cook), b) *R. integrifolia* subsp. *neomexicana* (Bob Sivinski), c) *R. integrifolia* subsp. *leedyi* (Welby R. Smith), d) *R. rhodantha* (Bryant Olsen), and e) *R. rosea* (Tero Laakso).

*Rhodiola integrifolia* is a characteristic plant of wet meadows, rocky cliffs, and scree slopes in the arctic and alpine tundra of the northern hemisphere. Like many other Crassulaceae, it is a low growing, tufted, dioecious perennial, and its erect stems, generally less than 12 cm (but up to 50 cm) tall, bear alternate, glabrous, sessile, succulent leaves, and possess a thick, fleshy rhizome [16]. The regal, wine-red flowers, with either 8-10 stamens or 5 pistils (depending on the sex of the plant), are organized in crown-like cymes, from which it derives its namesake King’s Crown (Figure 2a-c). All populations that have been studied have a base chromosome number of n=18 [17]. Finally, dispersal in this species may be either relatively limited or widespread, due to vegetative reproduction or winged seeds, respectively [17]. 

The species is traditionally divided into three subspecies (Figure 2a-c) based on morphology [14,16,17]. *Rhodiola integrifolia* Raf. subsp. *integrifolia* is short, with elliptical or ovate green leaves, usually red petals, and is patchily distributed across the arctic and alpine tundra from eastern Siberia [where it had been known as *R. atropurpurea* (Turcz.) Trautv. & C. A. Mey] to Alaska south to the Sierra Nevada in California and the southern Rocky Mountains of Colorado and New Mexico. In addition, Clausen [17] segregated tall (to 50 cm), robust populations as *Sedum integrifolium* subsp. *procerum* [=*R. integrifolia* subsp. *procera* (R.T. Clausen) H. Ohba], but this classification has not been maintained because its morphological variation overlaps with *R. integrifolia* subsp. *integrifolia* [16]. Leedy’s Stonecrop, *R. integrifolia* subsp. *leedyi* (Rosend. & J. W. Moore) H. Ohba, is tall (to 45 cm), with dark red petals that are (greenish-) yellow at the base, and has glaucous or blue-green leaves. It is federally listed as threatened because it is only known from a handful of isolated populations that inhabit dolomite cliffside seeps in Minnesota and New York. Finally, the New Mexican Stonecrop, *R. integrifolia* subsp. *neomexicana* (Britton) H. Ohba, differs in that it has yellow petals that are distally red (including the dorsal keel), narrow leaves, and grows on porphyritic rock at about 3500 m. It is considered imperiled because it is narrowly restricted to the Sierra Blanca Mountains of Lincoln Co., New Mexico, but is not federally listed.

In addition to *R. integrifolia*, two other species of the 60-90 accepted species of *Rhodiola* [18,19] occur naturally in North America: *R. rosea* L. (Figure 2d), and the North American endemic *R. rhodantha* (A. Gray) H. Jacobsen (Figure 2e). Like *R. integrifolia*, these species are perennials, with thick rhizomes, succulent leaves, and inflorescences arising from the leaf axils. Both *R. integrifolia* and *R. rosea* are dioecious and exhibit small, unisexual flowers in corymbose cymes. *R. integrifolia* was previously viewed as a subspecies of *R. rosea*, but has recently been elevated to species status [16,19,20,21,22] based on chromosomal differences (*R. integrifolia*, n = 18 [23,24,25], *R. rosea*, n = 11 [17,20,23]), and floral characteristics. The two species are readily differentiated based on the color of the corolla (petals of *R. rosea* are yellow versus red in *R. integrifolia*), floral merosity (*R. integrifolia* = 5-merous; *R. rosea* = 4-merous), and green (*R. integrifolia*) versus glaucous (*R. rosea*) leaves. Furthermore, species are non-overlapping in North America, with *R. rosea* being restricted to the eastern seaboard from Nunavut, Canada to Connecticut, USA. Also, *R. rhodantha* differs markedly from *R. integrifolia* and *R. rosea* in that its pinkish flowers are hermaphroditic, occurring in an elongate, raceme-like inflorescence. *Rhodiola rhodantha* occurs in the central and southern Rocky Mountains where it is sympatric with *R. integrifolia*. 

The evolutionary relationships among these three species have been continually debated, but must be determined in order to evaluate the extent to which *R. rosea* and *R. rhodantha* may have contributed to the genome and thus phylogeography of *R. integrifolia*. Based on chromosome counts, Uhl [23] hypothesized that either i) *R. rhodantha* (n = 7) was derived from a diploid ancestor of *R. rosea* (which he assumed to have a base chromosome number of 6) and *R. integrifolia* (n = 18) was a hexaploid of the n = 6 ancestor or ii) *R. integrifolia* (n = 18) arose as an allopolyploid of an ancestor of *R. rosea* (n = 11) and *R. rhodantha* (n = 7). The former hypothesis suggests that both *R. integrifolia* and *R. rhodantha* are more closely related than either is to *R. rosea*, while the latter predicts that *R. rhodantha* and *R. rosea* are more closely related than either is to *R. integrifolia*. Clausen [17] discussed these hypotheses and added that *R. integrifolia*, but probably not *R. rosea*, might have evolved from an ancestor with large, perfect, 5-merous flowers that also served as the progenitor of *R. rhodantha*. Recent phylogenetic analyses suggest that *R. rhodantha* and *R. integrifolia* are more closely related than either is to *R. rosea*, despite the greater morphological differences between *R. rhodantha* and *R. integrifolia. Rhodiola rosea* was placed within a different clade of Asian *Rhodiola* [15].

We set out to examine these infraspecific classifications and the speciation history in *R. integrifolia*, including the pattern, timing, and geography of genetic divergence. To accomplish this goal, we performed a phylogeographic analysis of all currently accepted subspecies of *R. integrifolia* employing a multi-locus genetic analysis in combination with ecological niche modeling. We adopted a two-pronged molecular approach; first, we performed a phylogenetic analysis using ptDNA on numerous species of *Rhodiola* to determine the closest relatives of *R. integrifolia*. Second, we estimated population genetic parameters, including effective population sizes, gene flow, and divergence times, for *R. integrifolia* using multi-locus nuclear data. Then we generated current and paleoecological niche models for *R. integrifolia* to assess variation in the environmental space occupied by the major clades identified in our phylogenetic analyses. Our findings for *Rhodiola* serve as a beacon to understand the history of divergence and degree of isolation among populations in an important North American artic-alpine zone. These data are necessary for evaluating conservation priorities and assessing how taxa may respond to ongoing climate change.

## Materials and Methods

### Molecular approach

We collected specimens from much of the range of *R. integrifolia* including the extremes of the species' distribution plus representative individuals of *R. rosea* and *R. rhodantha*. Specimens of *R. integrifolia* were sequenced from mainland Alaska and St. Lawrence Island in the Bering Sea, south along the Rocky Mountains to New Mexico and along the western coast in the Cascade and Sierra Nevada ranges (Figure 1b). Access to herbaria specimens from the eastern United States was limited, so those populations were undersampled (Figure 1c). Specimens from Siberia were unfortunately unavailable. A total of 35 specimens of *R. integrifolia* were sampled, representing 27 populations, including those from hypothesized refugial areas, and all known subspecies. Individuals were either collected from the field or from herbarium tissue (see Appendix S1 for Accessions). For the newly collected specimens, collecting permits were provided by the Arctic National Wildlife Refuge (permit #2006-S6) and the Alaska Maritime National Wildlife Refuge (permit 74500-06-015). Freshly collected individuals were preserved in Drierite (WA Hammond Drierite Co. Ltd., Xenia, OH, USA) and voucher specimens were pressed and mounted for curation in the Western Washington Herbarium (WWB; Appendix S1). We isolated genomic DNA from our tissue samples using the DNeasy Plant Extraction kit (Qiagen, Valencia, CA, USA), following the manufacturer’s recommended protocol. DNA was then sequenced from plastid and multiple nuclear loci and subjected to phylogenetic and population genetic analyses to determine the evolutionary history among subspecies and populations.

To place our focal taxa (*R. integrifolia* subsp. *integrifolia*, *R. integrifolia* subsp. *leedyi*, *R. integrifolia* subsp. *neomexicana*, *R. rhodantha*, and *R. rosea*) in the broader *Rhodiola* phylogeny, we performed an analysis using the plastid (pt) spacer region *trn*L-F, which has been broadly useful for resolving species level phylogenies of many angiosperm clades [26,27]. To accomplish this goal we supplemented our newly sequenced individuals of *R. integrifolia*, *R. rosea*, and *R. rhodantha* with additional GenBank sequences from 17 other species of *Rhodiola*, two additional sequences of *R. rosea*, and one outgroup, *Cotyledon campanulata* Marloth (see Appendix S2 for Accessions). We PCR amplified the pt *trn*L-F region as follows: each 25 μL reaction volume contained 2.25 mmol/L MgCl2, 0.02 mmol/L dNTPs, 0.05 mmol/L of each primer [26] (Table 1), 2.5 units of Invitrogen (Grand Island, New York, USA) Platinum Taq polymerase, Invitrogen 10X reaction buffer, and approximately 100 ng of genomic DNA and the thermal cycler profile was one cycle at 96°C for 5 min, 35 cycles at 96°C for 45 s, 48°C for 1 min, 72°C for 1 min 30 s, and one extension cycle at 72°C for 10 min. PCR products were sent to the PennState University Nucleic Acid Facility and to Functional Biosciences Inc. (Madison, WI) for enzymatic purification and Sanger sequencing in both directions.

**Table 1:** The anonymous nuclear locus primer sets (ALPS) for *Rhodiola*.

Locus	INDEL	bp	Primers
Rhod_1	3	381	TTTCCGAAATTCTTACCTCTCC
			TGAATCTGTGCCATTCAAGG
Rhod_2	0	535	TGATACGTTCGCCTCTGTCC
			ACGAGCCAGAAAAGTCAACG
Rhod_3	5	778	CCTCGTCTCATTCTCCATCC
			GGACAAGTGGTTGTGTTGTACC
Rhod_4	3	463	TGAAAACACGCTAAGGAGAGG
			GCATGCATCCATTCAATCC
Rhod_5	0	671	GAGAAAATCTGGTGCAGAGG
			GAAGCACATCGACATCAGG
*trn*L-F	5	296	ATTTGAACTGGTGACACGAG
			GGTTCAAGTCCCTCTATCCC

The number of INDELS (ID) that were coded as simple binary sites, the length of the locus (bp) following cutting as determined by recombination in RDP3, and the forward and reverse primers used for the ALPS and the plastid [26] are given.

We generated a multi-locus nuclear DNA (nDNA) dataset of anonymous non-coding loci for estimating the evolutionary history within *R. integrifolia*. The use of multiple nuclear loci in systematic studies greatly improves estimates of species trees and the demographic history of constituent taxa [28]. Non-coding markers were chosen as the basis for our phylogenetic and population genetic analyses because they are i) less likely to be under selection than coding genes, which would confound coalescent-based parameter estimation and ii) more likely to harbor informative nucleotide variation on the shallow timescales relevant to infraspecific studies. Further, our method of identifying loci allowed us to rapidly and randomly describe more non-coding loci and primers than could be obtained from the literature. 

Our approach to designing the anonymous locus primer sets (ALPS) for plants was a modification of that described by Galbreath et al. [29] based on Jennings et al. [30], using Invitrogen’s (Carlsbad, CA, USA) TOPO® Shotgun Subcloning Kit. In brief, we i) extracted > 6 micrograms of genomic DNA from leaf tissue of a single representative specimen of *R. integrifolia* (WWB accession no. 22742) using Qiagen’s DNeasy® Plant Extraction kit, ii) sheared DNA using a nebulizer, iii) blunt-end repaired the fragments, iv) electrophoretically separated DNA fragments on a 0.8% TAE gel, v) extracted fragments ranging from 1 to 1.4 kilobase (kb) in length using Qiagen’s QIAquick® Gel Extraction Kit, vi) added an adenine overhang for ligation into Invitrogen’s pCR®2.1-TOPO® TA vector and insertion into OneShot® TOP10 Competent cells, vii) grew cells on ampicillin-treated plates, and viii) screened colonies using standard blue-white screening. From the plates, we picked and lysed 32 colonies and used the lysate as template in PCR and sequencing reactions using the vector based primer M13. Vector sequence was trimmed from our contigs using Sequencher® v4.8 (Gene Codes Corp, Ann Arbor, MI, USA). To select the appropriate loci for use as ALPS, we ran blastn (nucleotide-nucleotide BLAST) and blastx (translated nucleotide-protein BLAST) searches against the nucleotide databases of the National Center for Biotechnology Information. We discarded DNA fragments matching organelle or protein coding sequences. Furthermore, we removed sequences that had long open reading frames. Fifteen sequences were selected for designing ALPS and primer pairs were optimized in Primer3-web 0.4.0 [31]. These primer sets were tested in temperature gradient PCR reactions to optimize annealing temperatures and to screen for non-target amplification. Ultimately, five ALPS reliably produced clean PCR products across all subspecies of *R. integrifolia* and were chosen for sequencing (Table 1). It is important to note that because ALPS were designed specifically for *R. integrifolia*, their utility with other taxa decreased with the degree of evolutionary relatedness. Specifically, the amplification and sequencing of ALPS worked well for all of our *R. integrifolia* samples, but less so for the distantly related *R. rhodantha* and *R. rosea*. 

In developing the nDNA dataset for *R. integrifolia*, our goal was to maximize the number of loci that worked across all populations and subspecies of *R. integrifolia* rather than the number of individuals because parameter estimation in coalescent analyses improves greatly by increasing the number of independent genetic markers [32]. Therefore, we selected a subsample of individuals that represented *R. integrifolia* subsp. *integrifolia* across the regions (Alaska N = 5, Pacific Northwest N=2, Sierra Nevada N=18, Southern Rocky Mountains N=6 [including *R. integrifolia* subsp. *neomexicana* and *R. integrifolia* subsp. *procera*]), *R. integrifolia* subsp. *leedyi* (*N* = 4), *R. rhodantha* (*N* = 2), and *R. rosea* (*N* = 7). Nuclear loci were amplified in 20 μl volume reactions, with final reagent concentrations of 1.5 mM MgCl2, 0.5 μM primers, 0.4 mM dNTPs, 0.5 U Taq polymerase, and approximately 5 ng/μl template DNA. All reactions included a 3 min. initial denaturation (94°C), 30 cycles of 30 sec denaturation (94°C), 30 sec annealing (55°C), and 1 min. extension (72°C), and a final 10 min. extension (72°C). All PCR products were cloned using TOPO® TA cloning kits and approximately 10 colonies per individual were used as PCR templates and sent to the PennState University Nucleic Acid Facility and to Functional Biosciences Inc. (Madison, WI) for enzymatic purification and Sanger sequencing in both directions. By taking this approach, we hoped to sample the haplotypes within an individual and thus improve our estimates of the genetic variation within each population cluster (as defined by STRUCTURE, see below).

In preparation for our analyses, all ptDNA and nDNA sequence contigs were assembled and edited using Sequencher® v4.8, aligned using ClustalX 2.0 [33], and alignments were manually edited in MacClade 4.08a [34]. We endeavored to include only orthologs in our analyses, to avoid the "perils of paralogy," such as inferring incorrect gene trees, divergence times, and population sizes [35]. For quality control, we identified paralogous sequences as those with ambiguous alignment or differences in length when compared with other clones within the same individual or across individuals and culled those sequences from our dataset. Furthermore, we manually coded INDELs as simple binary characters following Simmons and Ochoterena [36], and excluded all sites of ambiguous alignment from further analyses. 

As a final quality control step for our coalescent-based analyses, we tested each locus for recombination and either removed putative recombinant individuals or reduced the sequence length to the largest region that excluded recombinants. Recombination events confound coalescent analyses that explicitly assume no intralocus recombination by influencing the estimates of demographic parameters [37]. Thus, avoiding recombinants is essential in estimating the historical demography of a species. To test for recombination, we used the RDP [38], MAXCHI and CHIMAERA [39], and GENECOV [40] algorithms implemented in the software package RDP3 [41]. It is generally assumed that recombination is lacking for plastid markers, and is much more likely to encounter a recombinant within nuclear loci. Moreover, because plant nuclear genomes may contain multiple copies of any given gene, the opportunity for recombination in the nuclear genome is much higher [42]. By using the four aforementioned tests of recombination, we were relatively confident in our ability to identify and remove recombinants from downstream analyses. 

Using DNASP v. 5 [43], we calculated haplotype diversity (*h*), the number of segregating sites (θ_S_) and nucleotide diversity (π), and performed neutrality tests by comparing Tajima’s D [44] and Ful and Li’s D* and F* [45] statistics to 1000 coalescent simulations of a large, neutrally evolving population.

We then conducted maximum likelihood (ML) analyses in GARLI v0.96 [46] on all loci separately to infer the phylogeny. We performed separate phylogenetic analyses on each of the plastid *trn*L-F and five nDNA loci using the locus-specific DNA substitution model estimated in jModeltest 0.1.1 [47] (Table 1). In GARLI, we determined the best tree for each locus from ten replicate runs and then assessed branch support based on 200 bootstrap replicates (with five tree searches per replicate). For this, and further analyses we isolated the ptDNA and nDNA datasets in separate analyses because organelle and nDNA may have evolved under different population histories [29,48]. Phylogenies were archived in TreeBASE (http://purl.org/phylo/treebase/phylows/study/TB2:S14743).

The program STRUCTURE 2.3.2 [49] was applied to our multi-locus nDNA dataset to estimate the number of discrete population clusters, rather than relying on pre-defined population structure based on geography or taxonomic descriptions of subspecies. Each haplotype was coded as an allele. For these analyses, we used the admixture model of ancestry and both the correlated [50] and uncorrelated allele frequencies models, running separate analyses for all values of *K* (number of population clusters) between 1 and 7 (accounting for the overall number of disjunct regions and subspecies sampled). After a burn-in of 100,000 generations, we ran the analysis for an additional 100,000 generations and repeated them twice to confirm reproducibility.

The phylogenetic relationships among the four major population clusters of *R. integrifolia* (assigning individuals to regional groups based on the output from the STRUCTURE analyses), and *R. rhodantha*, using *R. rosea* as an outgroup (as determined by our ptDNA analyses of relationships within *Rhodiola*) were inferred in the program BEAST 1.6.2 [51], as implemented using *BEAST [52]. In doing so, all five of the nuclear loci were used in a single analysis. This coalescent-based approach produces more reliable multi-locus estimates of species trees and divergence times because alleles are not arbitrarily linked among loci as they might be in concatenated analyses [53]. We applied the Yule tree prior, given that we were estimating the relationships among several species and subspecies [51], and allowed rates to vary among loci. The ptDNA and nDNA analyses were run for 100 and 500 million generations, respectively, with 10% of each run discarded as burn-in. Stationarity was determined by examining parameter trend plots and effective sample size (ESS) values (all > 200) using TRACER 1.5 [54]. All analyses were repeated three times using different random seeds to confirm convergence of parameter estimates. To calculate divergence times, we used two different estimates of mutation rate: i) μ based on *trn*L-F 8.24e-9 substitutions/site/year calibrated from fossils of *Aichryson* Webb & Berthel. (Crassulaceae) [55] and ii) a general μ for plant autosomal genes of 1.1e-8 substitutions/site/year [56,57]. The resulting dates should be viewed with caution, given that i) *Aichryson* is distantly related to *Rhodiola* and ii) the autosomal mutation rate may be different from that of non-coding loci. 

The history of population size and gene flow was quantified simultaneously based on the nDNA dataset using the Bayesian formulation of LAMARC 2.1.6 [58]. For the demographic analyses, we applied the locus-specific models of nucleotide evolution from jModeltest [47], the estimates of relative mutation rates among loci from *BEAST, retained the default priors for θ (shape = logarithmic, upper bound = 10, lower bound = 1e-05) and migration (*M*), and adjusted the prior on the growth parameter (g) to a uniform distribution spanning -1000 to 2000. Final analyses were run for 100 million generations following 20 million generations of burn-in, sampling every 5000 generations. We assessed parameter trend plots, ESS values, and the shape of posterior distributions using TRACER to confirm stationarity and three separate runs were conducted to confirm repeatability of our results.

### Ecological niche models

Based on our molecular analyses, we evaluated differences in the distribution occupied by the two major *R. integrifolia* clades identified in our analyses–i.e., Beringia and the Southern Rocky Mountain Refugium (SRMR; excluding *R. rhodantha*; see results below)-by generating ecological niche models. First, the potential distribution of each clade was estimated based solely on occurrence records within that clade and then those were compared to visualize discrepancies in the capacity of records from one clade to predict the overall distribution of the lineage. Then, principle components analysis (PCA) and tests of niche equivalency and similarity were used to assess differences in the environmental space occupied by each clade. Our ecological niche models were built using georeferenced herbaria records (Figure 1a-c, Appendix S3) and CRU TS 2.1 climate data for the period 1971-2000 at its native 0.5° resolution [59]. The Beringia clade consisted of all records for all subspecies of *R. integrifolia* except those located in Colorado and New Mexico (n = 272 with one record used per pixel). The SRMR clade was composed of all records for Colorado and New Mexico (n = 39).

Predictor variables were selected using the random forest (RF) algorithm [60] based on recommendations by Strobl and colleagues [61]. Six variables were selected from an initial group of 44, based on RF output and Spearman’s correlation values (variables correlated at rho values ≤ |0.8|): annual temperature range (maximum temperature of the warmest month minus minimum temperature of the coldest month); mean diurnal range for summer and winter (mean of the maximum temperature minus minimum temperature of each month for June–August and December–February); summer minimum temperature (mean of minimum temperatures for June–August); precipitation of the wettest month; and cumulative spring snowpack (sum of snowpack for March–May). These variables correspond to climatic factors that are especially relevant to arctic-alpine plants [62].

An ensemble modeling approach was used to build ecological niche models. Ensemble modeling is based on the idea that different combinations of initial conditions, model algorithms, and general circulation models (GCMs) represent alternate possible states of the system being modeled [63]. When these are combined using consensus methods (such as the mean of all models), they can form a more accurate projection, outperforming single models [64]. For temporal projection, the two greatest sources of uncertainty in SDM models are the choice of model algorithm and the GCM used [65]. For this reason, the use of single algorithms and GCMs to project changes in species distributions has been criticized, especially for paleodistribution modeling [66]. In this study, we use an ensemble of multiple model algorithms and GCMs. 

Modeling was conducted with the BIOMOD package [67] in R v. 2.13.1 [68] and Maxent, v. 3.3.3 [69]. Eight model algorithms were used: generalized linear models, generalized additive models, multivariate adaptive regression splines, classification tree analysis, flexible discriminant analysis, generalized boosted models, random forest, and maximum entropy (Maxent). All models except Maxent were run using R. For all models the entire background of the study area was used to create pseudo-absence data, with these data weighted to maintain the number of presence records equal to the number of background records (prevalence = 0.5).

Model performance was assessed using ten bootstrapped replicates for each algorithm, partitioning the data into 70% for training and 30% for verification. The area under the curve of the receiver operating characteristic (AUC [70]) was averaged across replicates and used to determine if models should be removed from the ensemble. Models with mean AUC > 0.7 were determined to be useful and were kept in the consensus analysis. Final models were built using 100% of the available data [71]. Model probabilities were converted to presence/absence predictions using two thresholds: minimizing the absolute value of sensitivity minus specificity, and the mean probability value across model output. Both of these methods have been shown to perform well in threshold comparisons [72]. Current distributions represented the percentage of models voting “suitable habitat” for a given pixel based on 16 model votes (eight algorithms x two threshold methods). Final maps for each clade/species were based on 75% or more of the models voting suitable habitat for a given pixel.

Paleodistribution models were built using the combined *R. integrifolia* records (all individuals from both the Beringia and SRMR clades). Models were calibrated and evaluated as above and then projected onto climate data from the Mid-Holocene (6,000 years before present), the Last Glacial Maximum (LGM, 21,000 years before present), and the Last Interglacial (LIG, 124,000 years before present), which were assumed to serve as proxies for glacial and interglacial stages throughout the Pleistocene. Paleoclimate data were interpolated from their native resolution to 0.5° resolution using ordinary cokriging in ArcMap [73] using the CRU TS 2.1 climate data as the secondary data set. Mid-Holocene and LGM climate data were derived from three GCMs available through the Paleoclimate Modeling Intercomparison Project Phase 2 [74]: CCSM 3 [75], HadCM3 [76], and MIROC 3.2 [77]. Data for the LIG were provided through the HadCM3 model. Paleodistributions for the Mid-Holocene and LGM represent consensus across these three GCMs. The final refugial map was derived by assessing the probability of suitable habitat in each pixel across all four time slices (including the current distribution). The refugial map indicates pixels classified as suitable habitat across these time slices based on two thresholds for suitable habitat, 50 and 75%. The LGM ice layer [78] was overlaid to exclude potential suitable habitat under the ice sheet and create the final map of potential refugia. 

Principle components analysis (PCA) was used to evaluate the niche space occupied by the two focal clades of *R. integrifolia* based on climate variables (as described above) located by georeferenced herbarium samples. PCA was conducted in the context of the available environmental space, as recommended by Broennimann et al. [79]. Climate data were centered and standardized prior to PCA analysis; analyses were conducted in R using the ade4 package [80]. Overlap between the two clades in niche space was calculated using the *D* metric [79], which ranges between 0 (no overlap) and 1 (full overlap); randomization tests were used to assess niche equivalency and similarity. The null hypothesis for niche equivalency is that the clades have equivalent niches, while the null hypothesis for niche similarity is that the clades have retained niche similarity. Visualization of PCA analysis and calculation of *D*, equivalency and similarity were conducted with R code provided by O. Broennimann (Univ. Lausanne; further description of these tests can be found in [79]). 

## Results

**Figure 3:**
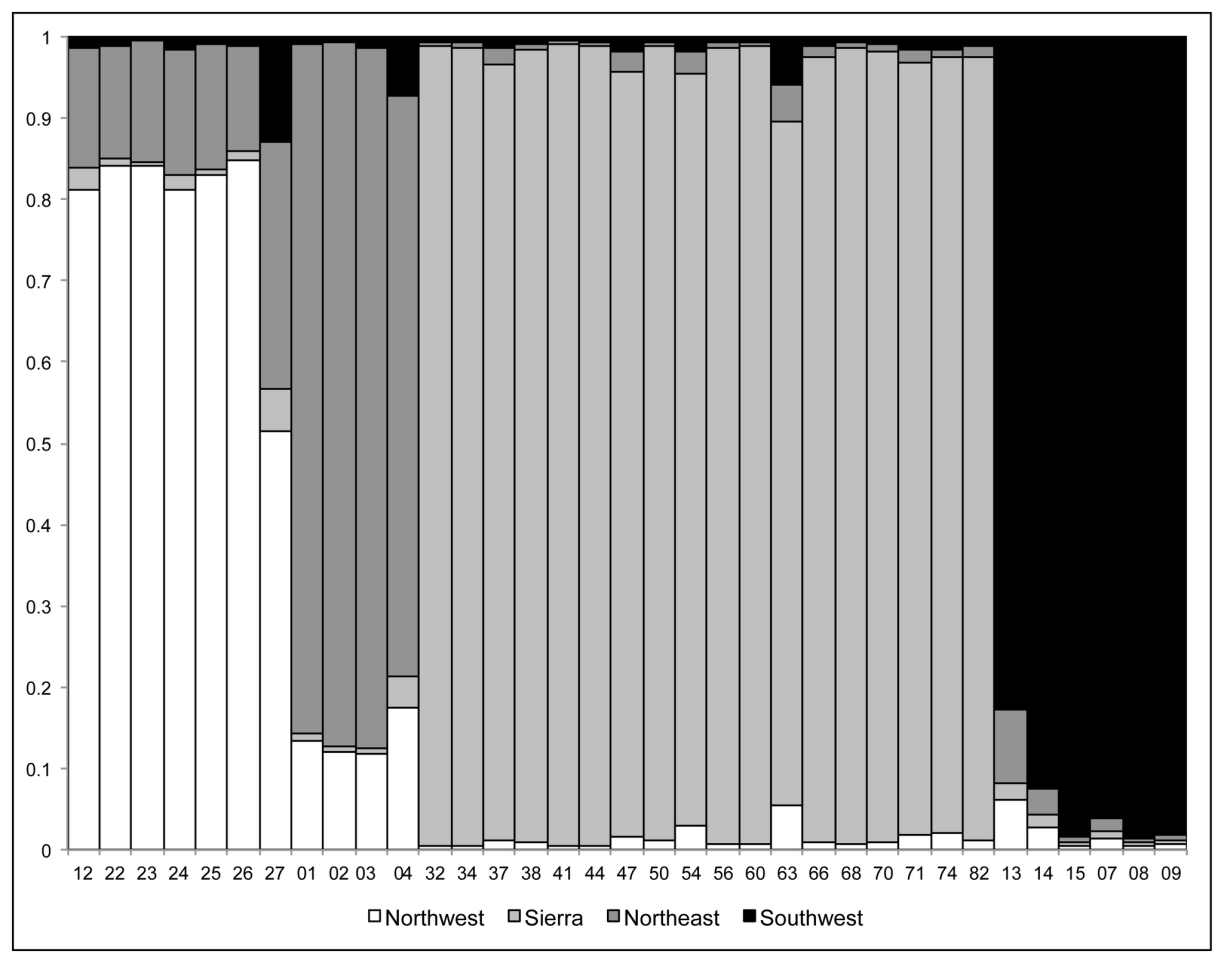
Population clustering results from the STRUCTURE analysis for *Rhodiola integrifolia*. The four cluster model (*K* = 4) shown was the most likely population model (mean ln likelihood = -352.2). The different fill shades denote separate population clusters as labeled. The vertical bars represent the proportion of the estimated cluster membership for each individual. Sample numbers are given along the bottom axis.

After culling sequences based on the RDP analyses of recombination, we included a total of 46 new *trn*L-F plastid (Genbank accessions KF681727 - KF681773) and 203 nDNA (among 5 loci; Genbank accessions KF681522 - KF681726) sequences of *R. integrifolia* and related species (Table 2). An excess of rare alleles was detected by the tests of neutrality across most of the loci (Table 2), which is indicative of population expansion, given that these loci are non-coding [44]. Positive growth (g) was also determined over all loci for each population cluster in Lamarc (Table 3). Tests of population association in STRUCTURE of the five nDNA loci showed a non-random distribution of haplotypes, revealing that a *K*=4 population model was the most likely (mean ln likelihood = -352.2), with the samples clustering as follows: Northwest = islands in the Bering Sea, arctic Alaska, Washington, and Montana; Northeast = Minnesota and New York (defined by *R. integrifolia* subsp. *leedyi*); Sierra = Sierra Nevada mountain range in California; and the Southwest = Colorado and New Mexico (Figure 3). 

**Table 2:** Locus-specific substitution model information for nuclear and plastid markers used for *Rhodiola*.

Locus	N	Nh	*h*	θ_S_	π	D	D*	F*	Model	Rate
Rhod_1	48	48	1.0000	0.141	0.096	-1.140	-2.542^a^	-2.384^a^	GTR+G	1
Rhod_2	29	27	0.9926	0.033	0.144	-2.068^a^	-3.431^a^	-3.515^a^	TPM1uf+G	0.291
Rhod_3	29	25	0.9877	0.022	0.151	-1.147	-2.200^b^	-2.188^b^	HKY+G	0.178
Rhod_4	29	27	0.9926	0.033	0.224	-1.240	-1.590	-1.742	TPM3uf+I+G	0.586
Rhod_5	68	66	0.9987	0.099	0.334	-2.280^a^	-5.342^a^	-4.854^a^	HKY+G	0.729
*trn*L-F	67	20	0.8019	0.023	0.008	-2.211^a^	-3.775^a^	-3.825^a^	TIM1+I+G	NA

The number of sequences (N) used in this study are given. From DNASP, the number of haplotypes (Nh), haplotype diversity (h , the per site measure of theta based on segregating sites (θ_S_) and nucleotide diversity (π) and test of neutrality through Tajima’s D, and Fu and Li’s D* and F* are shown where **a** denotes P<0.05 and **b** represents P<0.10. Also, the model of DNA substitution from JModeltest (Model), and the relative rate from * BEAST (Rate) are shown.

**Table 3:** Population genetic summary statistics for *Rhodiola integrifolia* from LAMARC based on the four population clusters determined in STRUCTURE.

				Migration (*M*) to:			
Population	θ	Ne (K)	g	Northwest	Northeast	Sierra	Southwest
Northwest (Beringia Clade)	1.386 (0.058-5.305)	15750 (659-60264)	144.6 (89.8-209.6)		157 (32-312)	97 (36-901)	6 (0-16)
Northeast (Beringia Clade)	2.280 (0.278-6.147)	25900 (3158-69829)	262.1 (181.9-374.1)	57 (8-858)		94 (33-970)	2 (0-12)
Sierra (Beringia Clade)	0.006 (0.003-0.590)	68 (34-6702)	187.4 (132.2-257.8)	115 (38-873)	179 (80-870)		6 (0-16)
Southwest (SRMR Clade)	0.134 (0.092-0.201)	1500 (1045-2283)	204.6 (150.2-289.6)	15 (0-50)	6 (0-47)	11 (0-35)	

The effective population size (Ne) is given in thousands (K) and was calculated from the estimate of theta (θ) using the equation θ = 4Neμ and the general autosomal mutation rate of plants of μ = 1.1e-8 substitutions/site/year x 2 years/generation. The estimated growth rate (g) and migration rates (*M*), representing the number of individuals per generation from the population clusters in the first column to the Northwest, Northeast, Sierra, and Southwest population clusters in the last four columns are also shown. 95% CI are given for each parameter in parentheses.

**Figure 4:**
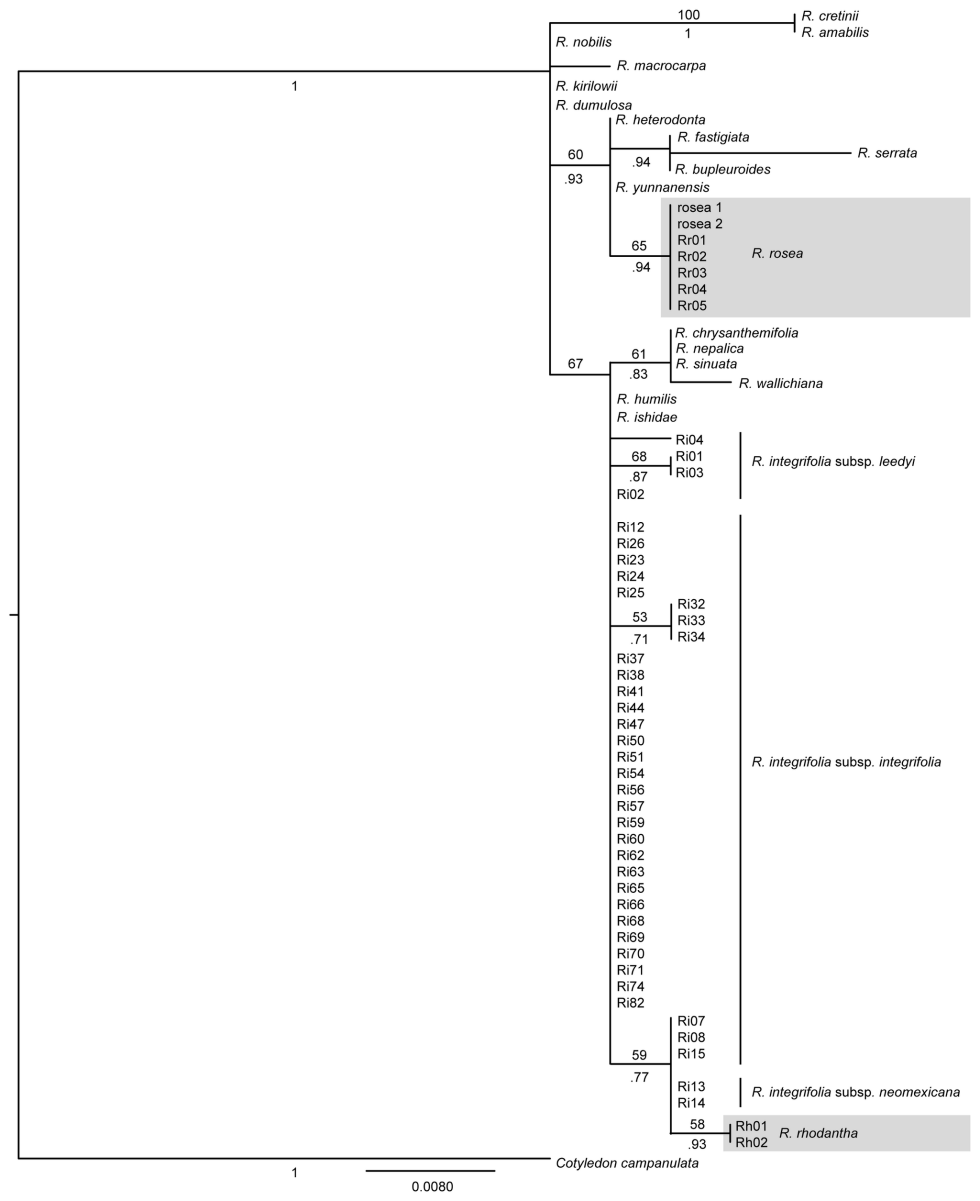
*trn*L-F phylogeny of *Rhodiola.* Maximum likelihood bootstrap values shown above branches; Bayesian posterior probabilities shown below branches. *Cotyledon campanulata* was used as an outgroup. *Rhodiola rosea* and *R. rhodantha* are blocked in gray and the subspecies of *R. integrifolia* are labeled.

The complete dataset of *Rhodiola* (and the outgroup *Cotyledon campanulata*) *trn*L-F sequences helped to estimate evolutionary relationships between *R. integrifolia*, *R. rhodantha*, and *R. rosea*. For the *trn*L-F plastid locus, the most appropriate model of DNA substitution was HKY+I, as determined by AIC in jModeltest (Table 2). Both the ML and Bayesian phylogenies suggested low (59% ML bootstrap/77 BPP) support for a close association between *R. rhodantha* and the Southwestern *R. integrifolia*, and importantly a moderately supported separation of *R. rosea* (Figure 4) from these two taxa (~ 65% ML bootstrap/94 BPP). 

**Figure 5:**
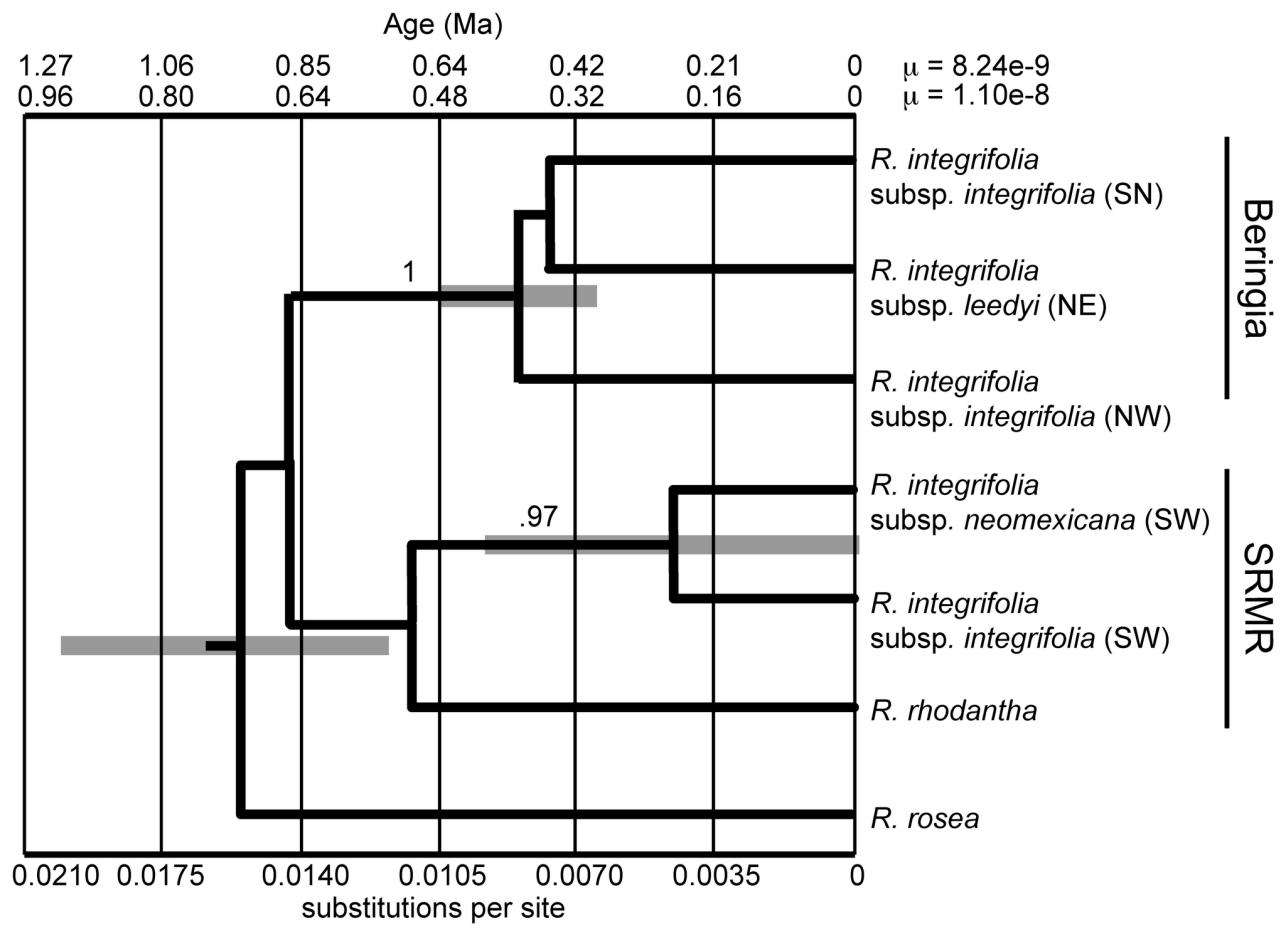
Phylogeny and divergence times based on five anonymous nuclear loci for populations *Rhodiola integrifolia*. Numbers above the branches represent Bayesian posterior probabilities for the node. The timescales at the top of the figure indicate divergence times as a function of substitutions per site (at the bottom) using rates based on the *Aichryson* fossil record in Crassulaceae (μ = 8.24e-9 [55]) and a general μ for plant autosomal genes of 1.1e-8 substitutions/site/year, respectively. Gray bars show the 95% highest posterior density for age estimates. The two main clades, Beringia and the Southern Rocky Mountain Refugium (SRMR), are labeled. Population clusters as determined by the STRUCTURE analyses are labeled as follows: Sierra (SN), Northeast (NE), Northwest (NW), and Southwest (SW).

The five nDNA loci further resolved relationships between *R. rosea, R. rhodantha*, and population clusters of *R. integrifolia*. The models of DNA substitution inferred from jModeltest, the relative rates from *BEAST (Table 2) and tree topologies for the individual loci (Figure S1) show the inherent variation in the stochastic nature of the evolutionary process for non-coding loci. Three important findings concerning the evolutionary history of *R. integrifolia* emerged from the *BEAST phylogenetic analyses of nDNA (Figure 5). First, *R. rosea* is distantly related to other North American *Rhodiola*, which corroborates findings from pt *trn*L-F. However, while *R. rosea* falls outside of the clade including the other two species, the time to most recent common ancestor (TMRCA) is still very recent, and within the Quaternary (~1 Mya, depending on the mutation rate). Second, *R. rhodantha* is closely related to *R. integrifolia* and hybridization may have occurred in the SRMR where the two species occur in sympatry. Third, *R. integrifolia* is deeply divided into two clades: one northern based in Beringia and the other in the Southern Rocky Mountain Refugium (SRMR) with little resolution within each clade. Very high Bayesian posterior probabilities (> 0.95) support a more recent (~700 kya; 660 kya or 880 kya depending on the mutation rate [Figure 5]) split between the Beringian and SRMR clades. Within each clade, the history of divergence is much more recent (~450 kya for Beringia versus only ~250 kya for the *R. integrifolia* subclades in the SRMR). 

Demographic analyses in LAMARC uncovered a history of isolation among longstanding population clusters (Table 3), as defined by STRUCTURE. Estimates of θ, and thus Ne, varied across populations. Effective population sizes were not related to the geographic distribution of a population cluster, with the Northeast harboring the largest θ (= 2.280) but over the smallest area, albeit with patchy microenvironments. In contrast, the Sierra had the smallest θ (= 0.006) but a much larger area and abundance of individuals. The two major clades, Beringia and the SRMR, have remained isolated from each other as exhibited by a signal of relatively very low migration (≤ 15, each of which includes 0 in the 95% CI) in comparison to within clade (57-179) gene flow.

**Figure 6:**
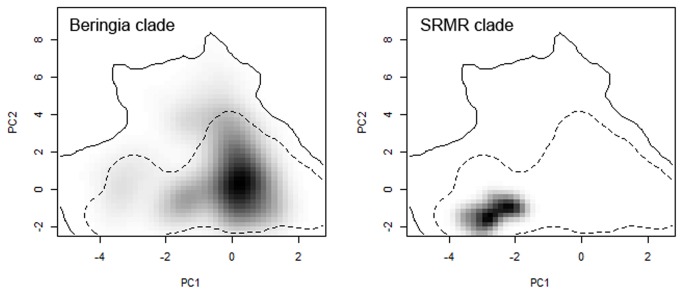
Niche for the Beringia (left) and Southern Rocky Mountain Refugium (SRMR, right) clades of *Rhodiola integrifolia* mapped in climatic space. Principle component (PC) axis 1 is defined by minimum summer temperature and summer mean diurnal range, while PC axis 2 is defined by spring snowpack and precipitation of the wettest month. Shading indicates the density of the PC scores for occurrence records in each clade. The contour lines indicate 50% (dotted) and 100% (solid) of the available environment. Note lack of niche overlap on PC1, and complete overlap on PC2.

**Figure 7:**
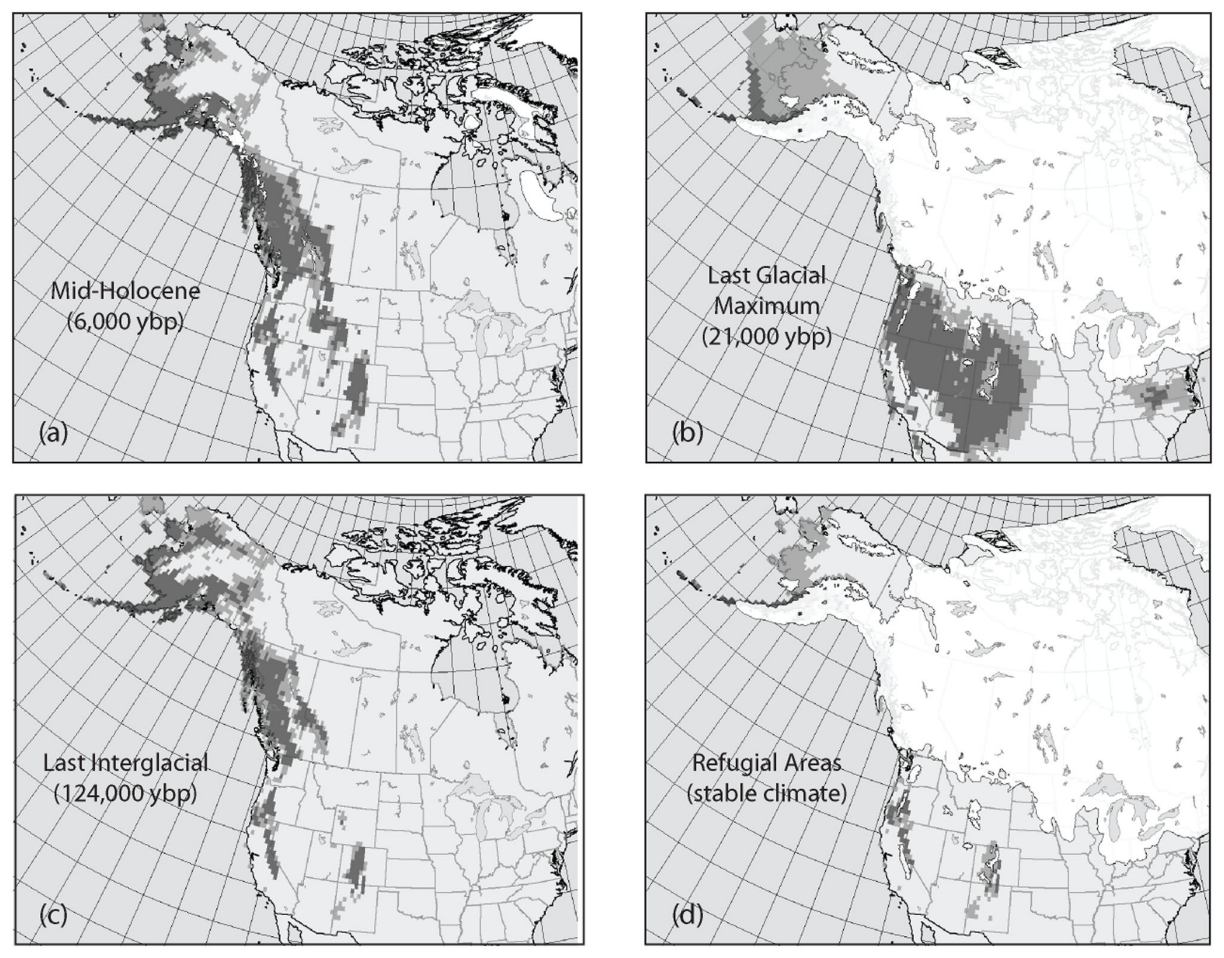
Paleodistribution maps for *Rhodiola integrifolia* at the (a) Mid-Holocene, (b) Last Glacial Maximum, and (c) Last Interglacial (ybp = years before present). Areas of stable climate over time are indicated in the (d) refugial map. Pixels with half of the ecological niche models voting suitable habitat are in light grey; those with 75% of the models voting suitable habitat are in dark grey.

Model performance for the ecological niche models showed that all models met the threshold for inclusion in the ensemble (AUC and sensitivity > 0.7), with all scores > 0.8, indicating very good model performance. For tests of niche space, niche overlap (*D*) between the Beringia and SRMR clades was 0.024 (p = 0.0198), rejecting the null hypothesis of niche equivalency between the two clades, and corroborating ecological niche models indicating low niche overlap (Figure 1d). Evaluation of the *D* statistic for niche similarity failed to reject the null hypothesis of retained niche similarity (p = 0.3168 for comparison of SRMR to Beringia, p = 0.7921 for comparison of Beringia to SRMR). This means that while the clades have different niches, they have retained similarities in their niches that are greater than expected by chance. The PCA plot (Figure 6) illustrated this, showing very little overlap on PC1 (defined by minimum summer temperature and summer mean diurnal range) and complete overlap on PC axis 2 (defined by spring snowpack and precipitation of the wettest month). The paleodistribution models uncovered a dynamic history of geographic shifts in available habitat space for *R. integrifolia* (Figure 7a-c), with three regions maintaining suitable habitat, and possibly serving as refugia for these plants, throughout the glacial-interglacial cycles (Figure 7d): Beringia in arctic Alaska, the Sierra Nevada, and the SRMR.

## Discussion

For arctic-alpine plants of North America, the climatic variability of the Quaternary promoted genetic divergence and allopatric speciation by isolating populations. In general, the fragmentation of populations across a species’ range occurred in two forms, dependent upon the climatic conditions [2]. During a cold, glacial stage several regional populations would have been isolated from one another in disjunct and potentially environmentally dissimilar refugia. Unlike the glacial refugia of lower elevation and lower latitude taxa, the terrain available to arctic-alpine species during an ice-age would have been vast, as the suitable habitat expanded down-slope to fill the lowlands and at the forefront of the ice-sheets, providing opportunities for local populations to grow, mix, and hybridize [3,7]. The available area of suitable habitat in regional refugia such as Beringia, the Driftless Area, the Sierra Nevada, and the Southern Rocky Mountains would have been much greater during a glacial episode than in a warm period. Thus, the glacial periods could be viewed as ‘good times’ for arctic-alpine plants: in which genetic variation increased, genetically diverse lineages were preserved, and genetic drift within regions was reduced. However, regions isolated by ice sheets would have diverged from one another in the absence of gene flow or in response to local selective pressures. The environment of the warm interglacials would have presented arctic-alpine taxa with different challenges associated with habitat fragmentation. Though the range of a species would not have been subdivided by ice sheets, and actually would have expanded across previously glaciated landscapes, patches of suitable habitat would have diminished in size, and in the case of the Driftless Area, likely been reduced to a few small cliffs with the appropriate microclimatic conditions [81]. As the climate warmed, populations in the west would have retreated upslope, becoming increasingly isolated from their neighbors on adjacent mountaintops. Moreover, along with the tundra habitat, populations would decrease in size, with many going locally extinct resulting in the potential loss of ancient lineages. This underscores the need for a combination of high elevation and high latitude environments for the preservation of these lineages through both cold and warm climatic periods. In North America, given the north-south orientation of the mountain chains, these sky islands would link previously isolated refugia along the cordilleran archipelago, from Beringia in the north all the way south along two contiguous cordilleras to southern California and New Mexico. However, this string of sky islands is not a continuous dispersal corridor, and mixing among refugia would have likely been limited. The consequence of this has been genetic divergence, and in the case of *R. integrifolia*, the derivation of endemic, including some presently threatened, taxa. 

### Relationships within *Rhodiola*

Our analyses have untangled the evolutionary history of *R. integrifolia*, helping shed light on taxa that are disjunctly distributed across the arctic-alpine biome. Our *trn*L-F phylogeny indicates that the North American *R. integrifolia* and *R. rhodantha* are more closely related to one another than either is to *R. rosea*. Indeed, *R. rosea* was placed in a separate clade of Asian taxa. Furthermore, this phylogeny indicated that some populations of *R. integrifolia* are more closely related to *R. rhodantha* than to other populations of *R. integrifolia*. The more rapidly evolving ALPS broadly confirmed our findings from these plastid analyses, in addition to clarifying the demographic history within *R. integrifolia*. Inferences from the five nDNA loci revealed two major clades, based in Beringia and the SRMR, that diverged ~ 700 kya (660 kya or 880 kya depending on the mutation rate [Figure 5]) and have remained isolated from each another. The close ties between the SRMR clade and *R. rhodantha*, with a divergence ~ 500 kya (or 660 kya depending on the mutation rate [Figure 5]), lends support to Clausen’s [17] and Guest's [15] hypotheses that *R. integrifolia* and *R. rhodantha* are closely related. The novelty from our study is that *R. integrifolia* has experienced a complex evolutionary history of isolation among multiple disjunct refugia since the mid-Pleistocene that gave rise to several lineages endemic to North America. 

The association between *R. integrifolia* and *R. rhodantha* evident in the tree topology could have arisen i) as the result of hybridization and introgression where the two species of *Rhodiola* overlap or ii) because *R. rhodantha* and the SRMR *R. integrifolia* clade are closely related. Though the chromosome counts and several characteristics (e.g., dioecy/hermaphrodite, flower color) differ between *R. rhodantha* and these southern *R. integrifolia*, they do share the common morphological feature of being taller plants in general, distinguishing the SRMR populations from *R. integrifolia* to the north. The hybridization scenario (i) likely involved an ancient split between *R. integrifolia* and *R. rhodantha* from a common ancestor as envisaged by Clausen [17]. Given the chromosomal and morphological differences between the species, the common occurrence of hybridization within the Crassulaceae [82], and the geographically sympatric distributions, the ancient split from a common ancestor [17] followed by more recent hybridization in the south between the two distinct species is a plausible scenario [15]. These analyses reveal that the evolutionary histories of *R. integrifolia* and *R. rhodantha* are tightly linked, but a more thorough analysis of members within this SRMR clade, including additional chromosome counts, are needed in order to more fully elucidate their history.

### Two geographically disjunct clades within *R. integrifolia*

We identified two major clades of *R. integrifolia* in this study, Beringia and SRMR, that diverged ~ 700 kya (660 kya or 880 kya depending on the mutation rate [Figure 5]). This immediately followed the Middle Pleistocene Transition (MPT) [83], and since then there has been extremely limited gene flow between the two clades. The MPT marked the transition towards an increase in the severity of glaciations, the emergence of the ~100-kyr glacial cycles (MPT beginning ~ 1250 kya [84]), and maximum glaciations and warmer interglacials becoming established at the end of the MPT ~700 kya [85]. *Sedum lanceolatum* also exhibited a dramatic phylogeographic break between the central and southern Rockies, across the low elevation and xeric habitat of the Wyoming Basin [86], during the same time-period (~600 kya [3,87]), even though it is not restricted to tundra habitats. Together, evidence from these species within the Crassulaceae suggests that the MPT had a profound impact on the distribution and diversification of arctic and alpine plants. 

Isolation in these two widely disjunct refugia is also independently supported by paleodistribution models, which indicate major refugial areas in Beringia, the Sierra Nevada Mountains and the southern Oregon Cascades, and the SRMR since the Last Interglacial (Figure 7d). Niche models based on current distributions of the Beringia and SRMR clades show that these models have a moderate ability to predict one another (Figure 1d) indicating that the clades continue to share some parts of their climatic niches, despite apparent genetic isolation between these lineages. This is also supported by PCA analysis (Figure 6), which shows very little overlap on PC axis 1 (defined by minimum summer temperature and summer mean diurnal range) and complete overlap on PC axis 2 (defined by spring snowpack and precipitation of the wettest month). Thus, based on the predictors we used in niche modeling, we see separation between the two clades based on minimum summer temperature and summer mean diurnal range. While statistical tests of the niches of the two clades indicate that they are not equivalent, the niches are more similar to each other than random expectation, supporting a likely role of geographic isolation in promoting genetic divergence in these taxa. 

#### The Beringia clade

 The northern Beringia subclade of *R. integrifolia* contains the majority of samples of *R. integrifolia* across its range, including *R. integrifolia* subsp. *integrifolia* from the Arctic south to the central Rocky Mountains and the Sierra Nevada of California and east to *R. integrifolia* subsp. *leedyi* in Minnesota and New York. An historic event approximately 350 kya led to a split between all three regions: Beringia, the Sierra Nevada, and the Driftless Area in the east. An examination of the current distribution maps (Figure 1) in conjunction with the levels of historic gene flow (Table 3) highlight the separation between these three regional population clusters. That said, only Beringia and the Sierra Nevada are evident as continual glacial-interglacial refugia (Figure 7d), and suitable habitat in the Driftless Area was all but gone during the interglacials, as is the case today. This clade harbors cryptic, endemic, and deep divergences, likely due to isolation of populations among several disjunct regions. 

Members of the Northwest subclade of *R. integrifolia* span a range from the Arctic coast of Alaska south to the Pacific Northwest of Washington and Montana. Across this distribution, the habitat varies extensively from rocky shores in the north to mountaintops in the south. Beringia is a well-documented refugium and corridor for transcontinental dispersal, north of the Pleistocene ice sheets, spanning from northeast Siberia across to northwestern North America [8,88], and is the likely refugium for the lineages of this Northwest clade of *R. integrifolia*. The central role that Beringia has played in the origin and preservation of the diversity of arctic-alpine plants in North America has been reviewed extensively [9,89,90,91,92,93,94,95]. With respect to *R. integrifolia*, this region has maintained a relatively high level of genetic diversity (Table 3), owing to its potential for long-term persistence of populations. Additional sampling may help to resolve the relationships among populations in the northwest.

Based on the ecological niche models, the Sierra Nevada mountain range of California at the southern end of the coastal cordillera had the strongest support of all potential refugial areas south of the ice sheets. The uplift of these granitic mountains, beginning approximately 4 Mya, and subsequent erosion by glaciers led to a topographically heterogeneous region that includes the highest point in the contiguous United States – Mt. Whitney (4,421 m). The varied topography of these mountains adds to the environmental heterogeneity responsible for the high levels of plant diversity and endemism within the California Floristic Province. Our analyses suggest that the Sierra subclade, the smallest (Table 3) of the three, is distinct from the other populations of *R. integrifolia* subsp. *integrifolia*, which is likely related to the effects of this environmental heterogeneity and isolation. The relatively low estimate for the Sierra Ne may be due to long distance colonization of this isolated region by only a few propagules. During the Pleistocene, montane glaciers blanketed most of the Sierra Nevadas [96,97,98,99], allowing high elevation taxa, such as *R. integrifolia*, to persist by moving downslope as has been suggested elsewhere [6,10,11,100,101,102,103,104]. These findings emphasize this region’s critical position as a refuge for arctic-alpine flora throughout both cold and warm climates.

In the Northeast, the Driftless Area of Wisconsin-Minnesota was ice-free during the last glaciation, as evinced by the lack of glacial drift across the region [13]. This geological evidence is supported by phylogeographic [6,100,101,102,103] and paleontological [105] studies. The Driftless Area differs from the other refugia in that it is not a mountainous landscape, but rather occurs with the bedrock of the Paleozoic Plateau that has been deeply dissected by streams [13]. Moreover, cool microhabitats (not the vast tracks of habitat available in other refugia during the interglacials) suitable for tundra plants have been maintained across this region due to the widespread development of karst formations and associated ice caves and cold air vents [106,107,108]. Interestingly, the population cluster with the fewest actual individuals, *R. integrifolia* subsp. *leedyi*, has the largest effective population size (Table 3), possibly owing to sampling individuals from distant, microclimate populations that have long been separate (e.g., Minnesota and New York). Indeed, lack of suitable habitat may have caused allopatric divergence within the Driftless Area, between microenvironments that harbor novel genetic diversity. The tree topology and population genetic parameters strongly support Leedy’s stonecrop as a separate subspecies. Like *R. integrifolia* subsp. *leedyi*, other arctic-alpine taxa endemic to the Driftless Area are threatened [109,110], highlighting the importance of this region and micro-environments for the maintenance of species’ diversity. 

#### The SRMR clade

 While the concept of refugia in North America south of the ice sheets is widely discussed in the literature [11,12], only a handful of studies have targeted plants distributed in the Southern Rockies [3,4,111,112]. These mountains house the second major clade uncovered in our analyses, consisting of the Colorado (*R. integrifolia* subsp. *integrifolia* [including *R. integrifolia* subsp. *procera*]) and New Mexico (*R. integrifolia* subsp. *neomexicana*) lineages of *R. integrifolia* and *R. rhodantha*. The population size of the Southwest lineages (the SRMR excluding *R. rhodantha*) is substantial, but lower than that of the northern populations (Table 3). Furthermore, the populations we sampled in Colorado and New Mexico are closely related as shown by our STRUCTURE analyses (inferred as one population cluster) and the recent TMRCA (Figure 5). Thus, alpine plants of the SRMR have persisted in isolation, with a high potential for genetic divergence from their close relatives in the mountains to the north. This appreciation for the role that southern mountain refugia have played in the origin and diversification of alpine plants is growing [113] and warrants further investigation. Though the alpine SRMR has received much less attention than its arctic counterpart (and other refugia south of the ice sheets for that matter), it has clearly been a major driver in the maintenance and diversification of alpine plants, particularly those endemic to North America [114], as exhibited in both our genetic data and ecological niche models. 

### Conservation implications

An improved understanding of systematics and the roles of refugia in maintaining and promoting diversity are central to understanding the process of speciation and informing conservation priorities [81]. The evolutionary history revealed by our multi-locus phylogeographic analyses shows that multiple genetically distinct and narrowly endemic *R. integrifolia* populations exist in North America, some of which are of conservation concern. The environmental differences among the disjunct refugia and the indication of diversification of ecological niches in these clades highlights the need to preserve these regions that harbor genetic variation and support processes of genetic divergence. Thus, these areas are important target areas for conservation under global change [115], and the demographic history of *R. integrifolia* should serve as a rationale for similar studies in other plant species.

Alpine species, with distributions defined by steep climatic gradients, are being disproportionately impacted by ongoing climate change [116,117,118,119,120]. In alpine and arctic systems, range shifts in response to climate change will likely result in increasingly fragmented and reduced habitat as species move higher in elevation and latitude, assuming that migration corridors remain intact. The consequence for many local populations will be extirpation as they run out of high elevation and high latitude habitat. Given the highly reduced and fragmented habitat available in the east and the scarcity of populations of *R. integrifolia* subsp. *leedyi*, this lineage is by far the most at risk from the changing environmental conditions associated with warming. Including refugial locations, especially those in the south or 'rear edge' [121], in conservation planning can help build evolutionary resiliency within populations, mediating extinction risks [122,123]. 

## Supporting Information

Appendix S1 **Specimen data for *Rhodiola integrifolia, R. rhodantha*, and *R. rosea* sequences generated in this study.**
(DOC)

Appendix S2 **GenBank accession numbers for data not generated in this study.**
(DOC)

Appendix S3 **Herbarium records for *Rhodiola integrifolia* (and all taxonomic synonyms) used in niche modeling (725 total).**
(DOC)

Figure S1 **Maximum likelihood trees for each of the 5 anonymous nuclear loci.** A. Rhod_1, B. Rhod_2, C. Rhod_3, D. Rhod_4, and 5. Rhod_5. Where possible, *R. rosea* was used as the outgroup, otherwise trees were rooted at the midpoint. All branch lengths are normalized to the scale bar showing 0.01 substitutions. The branches for *R. rosea* and *R. rhodantha* are labeled. Bootstrap values >70 are shown by an *.
(TIF)
