# Supplementary material for: Deep Genetic Divergence between Disjunct Refugia in the Arctic-Alpine King’s Crown, Rhodiola integrifolia (Crassulaceae)
Source: PLoS One. 2013 Nov 1;8(11):e79451. doi: 10.1371/journal.pone.0079451 (PMC3838311; doi:10.1371/journal.pone.0079451)
Supplement: Appendix S1 — Specimen data for Rhodiola integrifolia, R. rhodantha, and R. rosea sequences generated in this study. (DOC) [file pone.0079451.s001.doc]

Appendix S1. Specimen data for *Rhodiola integrifolia, R. rhodantha*, and *R. rosea* sequences generated in this study.

| Sample | Species/subsp. | Geographical group | Location | Lat. | Long. | Voucher |
| --- | --- | --- | --- | --- | --- | --- |
|  | *R. integrifolia* |  |  |  |  |  |
| Ri01 | *leedyi* | Northeast | USA: NY, Yates Cty. | 42.50 | -76.92 | DUL 456696 |
| Ri02 | *leedyi* | Northeast | USA: MN, Olmstead Cty. | 44.10 | -92.13 | DUL 456703 |
| Ri03 | *leedyi* | Northeast | USA: NY, Yates Cty. | 42.50 | -76.92 | DUL 520732 |
| Ri04 | *leedyi* | Northeast | USA: MN, Fillmore Cty. | 43.73 | -92.34 | DUL 787290 |
| Ri07 | *integrifolia* | Southwest | USA: CO, Ouray Cty., Red Mtn. Pass | 37.90 | -107.71 | WWB 22742 |
| Ri08 | *integrifolia* | Southwest | USA: CO, Ouray Cty., Red Mtn. Pass | 37.90 | -107.71 | WWB 22743 |
| Ri09 | *integrifolia* | Southwest | USA: CO, Ouray Cty., Red Mtn. Pass | 37.90 | -107.71 | WWB 22744 |
| Ri12 | *integrifolia* | Northwest | USA: MT, Madison Cty., Sphinx Mtn. | 45.17 | -111.49 | RMH 740006 |
| Ri13 | *neomexicana* | Southwest | USA: NM, Otero Cty., Sierra Blanca Pk. | 33.37 | -105.81 | NMC 13704 |
| Ri14 | *neomexicana* | Southwest | USA: NM, Otero Cty., Sierra Blanca Pk. | 33.37 | -105.81 | NMC 13704 |
| Ri15 | *integrifolia* | Southwest | USA: NM, Colfax Cty. | 36.83 | -105.22 | RMH 737944 |
| Ri22 | *integrifolia* | Northwest | USA: AK, St. Lawrence Is. | 63.42 | -170.40 | WWB 22749 |
| Ri23 | *integrifolia* | Northwest | USA: AK, ANWR | 70.06 | -145.49 | WWB 22750 |
| Ri24 | *integrifolia* | Northwest | USA: AK, ANWR | 70.06 | -145.49 | WWB 22751 |
| Ri25 | *integrifolia* | Northwest | USA: AK, ANWR | 70.06 | -145.49 | WWB 22752 |
| Ri26 | *integrifolia* | Northwest | USA: WA, Whatcom Cty., Mt. Baker | 48.82 | -121.83 | WWB 22753 |
| Ri27 | *integrifolia* | Northwest | USA: AK, St. Matthew Is. | 60.55 | -172.92 | ALA V158728 |
| Ri32 | *integrifolia* | Sierra | USA: CA, Nevada Cty., Donner Pass | 39.31 | -120.33 | WWB 22754 |
| Ri34 | *integrifolia* | Sierra | USA: CA, Nevada Cty., Donner Pass | 39.31 | -120.33 | WWB 22755 |
| Ri37 | *integrifolia* | Sierra | USA: CA, Eldorado Cty., Fallen Leaf Lk. | 38.87 | -120.08 | WWB 22756 |
| Ri38 | *integrifolia* | Sierra | USA: CA, Alpine Cty., Round Top Pk. | 38.67 | -120.01 | WWB 22757 |
| Ri41 | *integrifolia* | Sierra | USA: CA, Tuolumne Cty., Dardanelles | 38.40 | -119.87 | WWB 22758 |
| Ri44 | *integrifolia* | Sierra | USA: CA, Mono Cty., Gardisky Lk. | 37.96 | -119.25 | WWB 22759 |
| Ri45 | *integrifolia* | Sierra | USA: CA, Mono Cty., Gardisky Lk. | 37.96 | -119.25 | WWB 22760 |
| Ri47 | *integrifolia* | Sierra | USA: CA, Mono Cty., Virginia Pass | 38.07 | -119.33 | WWB 22761 |
| Ri50 | *integrifolia* | Sierra | USA: CA, Mono Cty., Lk. Genevieve | 37.55 | -118.88 | WWB 22762 |
| Ri54 | *integrifolia* | Sierra | USA: CA, Inyo Cty., Blue Lk. | 37.19 | -118.62 | WWB 22763 |
| Ri56 | *integrifolia* | Sierra | USA: CA, Inyo Cty., Table Mtn. | 37.18 | -118.59 | WWB 22764 |
| Ri60 | *integrifolia* | Sierra | USA: CA, Inyo Cty., Summit Lk. | 37.14 | -118.50 | WWB 22765 |
| Ri63 | *integrifolia* | Sierra | USA: CA, Inyo Cty., Kearsage Lk. | 36.76 | -118.38 | WWB 22766 |
| Ri66 | *integrifolia* | Sierra | USA: CA, Inyo Cty., Matlock Lk. | 36.76 | -118.36 | WWB 22767 |
| Ri68 | *integrifolia* | Sierra | USA: CA, Inyo Cty., High Lk. | 36.49 | -118.24 | WWB 22768 |
| Ri70 | *integrifolia* | Sierra | USA: CA, Inyo Cty., High Lk. | 36.49 | -118.23 | WWB 22769 |
| Ri71 | *integrifolia* | Sierra | USA: CA, Tulare Cty., Eagle Lk. | 36.42 | -118.60 | WWB 22770 |
| Ri74 | *integrifolia* | Sierra | USA: CA, Tulare Cty., Emerald Lk. | 36.60 | -118.68 | WWB 22771 |
| Ri82 | *integrifolia* | Sierra | USA: CA, Fresno Cty., Kaiser Pk. | 37.30 | -119.19 | WWB 22772 |
| Rh10 | *R. rhodantha* |  | USA: CO, Ouray Cty., Red Mtn. Pass | 37.90 | -107.71 | WWB 22745 |
| Rh11 | *R. rhodantha* |  | USA: CO, Ouray Cty., Red Mtn. Pass | 37.90 | -107.71 | RMH 763471 |
| Rr01 | *R. rosea* |  | Norway: Troms, Lenangstroya | 69.97 | 20.28 | WWB 22746 |
| Rr02 | *R. rosea* |  | Norway: Troms, Lenangstroya | 69.97 | 20.28 | WWB 22747 |
| Rr03 | *R. rosea* |  | Norway: Finnmark, Mageroya | 71.05 | 25.91 | WWB 22748 |
| Rr04 | *R. rosea* |  | Russia: Chukotka | N/A | N/A | RMH 330041 |
| Rr05 | *R. rosea* |  | Greenland | N/A | N/A | RMH 397668 |
| Rr06 | *R. rosea* |  | Russia: Chukotka, Koryakskoye Mtns. | 62.48 | -171.87 | ALA V115350 |
| Rr07 | *R. rosea* |  | Russia: Chukotka, Anadyr | 64.73 | 177.50 | ALA V114130 |
