# Supplementary material for: Deep Genetic Divergence between Disjunct Refugia in the Arctic-Alpine King’s Crown, Rhodiola integrifolia (Crassulaceae)
Source: PLoS One. 2013 Nov 1;8(11):e79451. doi: 10.1371/journal.pone.0079451 (PMC3838311; doi:10.1371/journal.pone.0079451)
Supplement: Appendix S2 — GenBank accession numbers for data not generated in this study. (DOC) [file pone.0079451.s002.doc]

Appendix S2. GenBank accession numbers for data not generated in this study.

*Cotyledon campanulata* (AY692293), *Rhodiola amabilis* (AB089760), *R. bupleuroides*  (AB089750), *R. chrysanthemifolia* (AB089752), *R. cretinii* (AB089761), *R. dumulosa* (AB089744), *R. fastigiata* (AB089749), *R. heterodonta* (AB089745), *R. humilis* (AB089755), *R. ishidae* (AB089751), *R. kirilowii* (FJ974038), *R. macrocarpa* (AB089759), *R. nepalica* (AB089754), *R. nobilis* (AB089758), *R. serrata* (AB089747), *R. sinuata* (AB089753), *R. wallichiana* (AB089757), *R. yunnanensis* (AB089748)
