# Supplementary material for: Deep Genetic Divergence between Disjunct Refugia in the Arctic-Alpine King’s Crown, Rhodiola integrifolia (Crassulaceae)
Source: PLoS One. 2013 Nov 1;8(11):e79451. doi: 10.1371/journal.pone.0079451 (PMC3838311; doi:10.1371/journal.pone.0079451)
Supplement: Appendix S3 — Herbarium records for Rhodiola integrifolia (and all taxonomic synonyms) used in niche modeling (725 total). (DOC) [file pone.0079451.s003.doc]

Appendix S3. Herbarium records for *Rhodiola integrifolia* (and all taxonomic synonyms) used in niche modeling (725 total). One herbarium record was used per pixel of climate data, resulting in a final data set of 311 records used for modeling.

| Species/subsp. | Location | Lat. | Long. | Voucher |
| --- | --- | --- | --- | --- |
| *Rhodiola integrifolia* | USA: CA, Inyo Cty. | 36.58 | -118.24 | SBBG70353 |
| *Rhodiola integrifolia* | USA: CA, Tulare Cty. | 36.60 | -118.68 | SBBG14376 |
| *Rhodiola integrifolia* | USA: CA, Inyo Cty. | 36.78 | -118.37 | SD70771 |
| *Rhodiola integrifolia* | USA: CA, Fresno Cty. | 36.89 | -118.65 | SBBG47899 |
| *Rhodiola integrifolia* | USA: CA, Mono Cty. | 37.50 | -118.25 | SD62250 |
| *Rhodiola integrifolia* | USA: CA, Mono Cty. | 37.59 | -119.01 | SBBG16275 |
| *Rhodiola integrifolia* | USA: CA, Mono Cty. | 37.92 | -119.22 | SBBG16384 |
| *Rhodiola integrifolia* | USA: CA, Tuolumne Cty. | 37.92 | -119.27 | SBBG16013 |
| *Rhodiola integrifolia* | USA: WA, Okanogan Cty. | 48.69 | -119.92 | WTU20904 |
| *Rhodiola integrifolia* | USA: WA, Okanogan Cty. | 48.93 | -120.21 | WTU376976 |
| *Rhodiola integrifolia* | USA: WA, Okanogan Cty. | 48.97 | -120.19 | WTU376974 |
| *Rhodiola integrifolia* | N/A | 54.68 | -163.18 | ALA45488 |
| *Rhodiola integrifolia* | N/A | 54.91 | -132.95 | ALA23474 |
| *Rhodiola integrifolia* | N/A | 55.08 | -162.03 | ALA46515 |
| *Rhodiola integrifolia* | N/A | 55.14 | -162.83 | ALA21129 |
| *Rhodiola integrifolia* | N/A | 55.34 | -160.43 | ALA146321 |
| *Rhodiola integrifolia* | N/A | 55.58 | -131.21 | ALA41378 |
| *Rhodiola integrifolia* | N/A | 55.71 | -133.56 | ALA8753 |
| *Rhodiola integrifolia* | N/A | 55.86 | -134.26 | ALA41922 |
| *Rhodiola integrifolia* | N/A | 55.88 | -134.25 | ALA23464 |
| *Rhodiola integrifolia* | N/A | 55.92 | -134.32 | ALA41960 |
| *Rhodiola integrifolia* | N/A | 56.23 | -133.58 | ALA8710 |
| *Rhodiola integrifolia* | N/A | 56.33 | -158.50 | ALA23483 |
| *Rhodiola integrifolia* | N/A | 56.90 | -158.06 | ALA46514 |
| *Rhodiola integrifolia* | N/A | 57.16 | -154.23 | ALA48372 |
| *Rhodiola integrifolia* | N/A | 57.20 | -153.18 | ALA46518 |
| *Rhodiola integrifolia* | N/A | 57.27 | -154.20 | ALA147552 |
| *Rhodiola integrifolia* | N/A | 57.45 | -153.19 | ALA145844 |
| *Rhodiola integrifolia* | N/A | 57.45 | -154.67 | ALA144615 |
| *Rhodiola integrifolia* | N/A | 57.45 | -154.58 | ALA145015 |
| *Rhodiola integrifolia* | N/A | 57.46 | -135.96 | ALA43197 |
| *Rhodiola integrifolia* | N/A | 57.56 | -156.03 | ALA48375 |
| *Rhodiola integrifolia* | N/A | 57.58 | -153.38 | ALA46516 |
| *Rhodiola integrifolia* | N/A | 57.70 | -153.88 | ALA4393 |
| *Rhodiola integrifolia* | N/A | 57.81 | -152.45 | ALA23469 |
| *Rhodiola integrifolia* | N/A | 57.91 | -152.33 | ALA46519 |
| *Rhodiola integrifolia* | N/A | 58.11 | -152.53 | ALA5612 |
| *Rhodiola integrifolia* | N/A | 58.20 | -157.38 | ALA48378 |
| *Rhodiola integrifolia* | N/A | 58.21 | -153.00 | ALA7961 |
| *Rhodiola integrifolia* | N/A | 58.23 | -152.66 | ALA5568 |
| *Rhodiola integrifolia* | N/A | 58.25 | -152.58 | ALA4797 |
| *Rhodiola integrifolia* | N/A | 58.30 | -134.40 | ALA44301 |
| *Rhodiola integrifolia* | N/A | 58.31 | -134.38 | ALA44304 |
| *Rhodiola integrifolia* | N/A | 58.41 | -134.54 | ALA9506 |
| *Rhodiola integrifolia* | N/A | 58.46 | -152.71 | ALA162077 |
| *Rhodiola integrifolia* | N/A | 58.55 | -161.76 | ALA48374 |
| *Rhodiola integrifolia* | N/A | 58.56 | -152.66 | ALA5794 |
| *Rhodiola integrifolia* | N/A | 58.60 | -159.96 | ALA23468 |
| *Rhodiola integrifolia* | N/A | 58.61 | -134.50 | ALA44303 |
| *Rhodiola integrifolia* | N/A | 58.63 | -153.78 | ALA60379 |
| *Rhodiola integrifolia* | N/A | 58.66 | -134.25 | ALA44302 |
| *Rhodiola integrifolia* | N/A | 58.86 | -158.75 | ALA48377 |
| *Rhodiola integrifolia* | N/A | 58.88 | -152.03 | ALA48373 |
| *Rhodiola integrifolia* | N/A | 58.95 | -158.48 | ALA48376 |
| *Rhodiola integrifolia* | N/A | 59.01 | -155.90 | ALA60319 |
| *Rhodiola integrifolia* | N/A | 59.07 | -161.89 | ALA89789 |
| *Rhodiola integrifolia* | N/A | 59.10 | -161.75 | ALA90001 |
| *Rhodiola integrifolia* | N/A | 59.10 | -155.71 | ALA82516 |
| *Rhodiola integrifolia* | N/A | 59.20 | -138.58 | ALA4469 |
| *Rhodiola integrifolia* | N/A | 59.28 | -158.61 | ALA4530 |
| *Rhodiola integrifolia* | N/A | 59.28 | -158.60 | ALA54119 |
| *Rhodiola integrifolia* | N/A | 59.28 | -158.55 | ALA54125 |
| *Rhodiola integrifolia* | N/A | 59.28 | -136.11 | ALA34081 |
| *Rhodiola integrifolia* | N/A | 59.30 | -158.53 | ALA54186 |
| *Rhodiola integrifolia* | N/A | 59.30 | -135.36 | ALA23481 |
| *Rhodiola integrifolia* | N/A | 59.39 | -138.10 | ALA74903 |
| *Rhodiola integrifolia* | N/A | 59.41 | -161.15 | ALA79163 |
| *Rhodiola integrifolia* | N/A | 59.43 | -138.93 | ALA51043 |
| *Rhodiola integrifolia* | N/A | 59.44 | -153.95 | ALA59716 |
| *Rhodiola integrifolia* | N/A | 59.53 | -150.46 | ALA51716 |
| *Rhodiola integrifolia* | N/A | 59.55 | -135.06 | ALA23480 |
| *Rhodiola integrifolia* | N/A | 59.58 | -151.30 | ALA51482 |
| *Rhodiola integrifolia* | N/A | 59.60 | -153.69 | ALA59715 |
| *Rhodiola integrifolia* | N/A | 59.60 | -135.14 | ALA74764 |
| *Rhodiola integrifolia* | N/A | 59.61 | -135.16 | ALA63642 |
| *Rhodiola integrifolia* | N/A | 59.71 | -154.90 | ALA51921 |
| *Rhodiola integrifolia* | N/A | 59.75 | -166.16 | ALA54603 |
| *Rhodiola integrifolia* | N/A | 59.75 | -161.25 | ALA54476 |
| *Rhodiola integrifolia* | N/A | 59.75 | -154.91 | ALA67802 |
| *Rhodiola integrifolia* | N/A | 59.83 | -154.70 | ALA67847 |
| *Rhodiola integrifolia* | N/A | 59.86 | -166.05 | ALA23462 |
| *Rhodiola integrifolia* | N/A | 59.86 | -160.08 | ALA7500 |
| *Rhodiola integrifolia* | N/A | 59.93 | -149.70 | ALA51404 |
| *Rhodiola integrifolia* | N/A | 59.96 | -158.53 | ALA54366 |
| *Rhodiola integrifolia* | N/A | 60.00 | -166.00 | ALA23488 |
| *Rhodiola integrifolia* | N/A | 60.05 | -148.06 | ALA5092 |
| *Rhodiola integrifolia* | N/A | 60.16 | -159.20 | ALA55672 |
| *Rhodiola integrifolia* | N/A | 60.16 | -149.63 | ALA57507 |
| *Rhodiola integrifolia* | N/A | 60.20 | -166.93 | ALA55229 |
| *Rhodiola integrifolia* | N/A | 60.20 | -148.30 | ALA9033 |
| *Rhodiola integrifolia* | N/A | 60.25 | -143.08 | ALA60170 |
| *Rhodiola integrifolia* | N/A | 60.36 | -149.01 | ALA57630 |
| *Rhodiola integrifolia* | N/A | 60.38 | -166.18 | ALA55276 |
| *Rhodiola integrifolia* | N/A | 60.38 | -166.16 | ALA23459 |
| *Rhodiola integrifolia* | N/A | 60.40 | -172.70 | ALA23470 |
| *Rhodiola integrifolia* | N/A | 60.44 | -143.89 | ALA141224 |
| *Rhodiola integrifolia* | N/A | 60.48 | -149.75 | ALA23473 |
| *Rhodiola integrifolia* | N/A | 60.50 | -173.00 | ALA54883 |
| *Rhodiola integrifolia* | N/A | 60.55 | -172.92 | ALA130609 |
| *Rhodiola integrifolia* | N/A | 60.61 | -165.25 | ALA55341 |
| *Rhodiola integrifolia* | N/A | 60.71 | -152.66 | ALA142747 |
| *Rhodiola integrifolia* | N/A | 60.76 | -148.31 | ALA58378 |
| *Rhodiola integrifolia* | N/A | 60.78 | -153.86 | ALA32435 |
| *Rhodiola integrifolia* | N/A | 60.91 | -159.86 | ALA192066 |
| *Rhodiola integrifolia* | N/A | 60.96 | -145.00 | ALA23463 |
| *Rhodiola integrifolia* | N/A | 60.98 | -153.84 | ALA47241 |
| *Rhodiola integrifolia* | N/A | 61.00 | -159.95 | ALA7456 |
| *Rhodiola integrifolia* | N/A | 61.05 | -159.80 | ALA64573 |
| *Rhodiola integrifolia* | N/A | 61.06 | -149.83 | ALA61864 |
| *Rhodiola integrifolia* | N/A | 61.11 | -155.60 | ALA31661 |
| *Rhodiola integrifolia* | N/A | 61.13 | -145.75 | ALA61569 |
| *Rhodiola integrifolia* | N/A | 61.13 | -145.73 | ALA23461 |
| *Rhodiola integrifolia* | N/A | 61.18 | -159.74 | ALA42096 |
| *Rhodiola integrifolia* | N/A | 61.20 | -146.31 | ALA61487 |
| *Rhodiola integrifolia* | N/A | 61.26 | -149.53 | ALA16041 |
| *Rhodiola integrifolia* | N/A | 61.35 | -152.83 | ALA59717 |
| *Rhodiola integrifolia* | N/A | 61.36 | -148.91 | ALA59718 |
| *Rhodiola integrifolia* | N/A | 61.40 | -149.06 | ALA62127 |
| *Rhodiola integrifolia* | N/A | 61.41 | -165.41 | ALA23482 |
| *Rhodiola integrifolia* | N/A | 61.43 | -165.45 | ALA64975 |
| *Rhodiola integrifolia* | N/A | 61.53 | -149.90 | ALA62468 |
| *Rhodiola integrifolia* | N/A | 61.66 | -149.10 | ALA62784 |
| *Rhodiola integrifolia* | N/A | 61.71 | -166.13 | ALA64813 |
| *Rhodiola integrifolia* | N/A | 61.76 | -158.13 | ALA64405 |
| *Rhodiola integrifolia* | N/A | 61.78 | -149.26 | ALA63001 |
| *Rhodiola integrifolia* | N/A | 61.81 | -149.20 | ALA63099 |
| *Rhodiola integrifolia* | N/A | 61.85 | -165.58 | ALA65012 |
| *Rhodiola integrifolia* | N/A | 61.86 | -154.55 | ALA64060 |
| *Rhodiola integrifolia* | N/A | 61.98 | -149.16 | ALA63135 |
| *Rhodiola integrifolia* | N/A | 62.18 | -165.58 | ALA64850 |
| *Rhodiola integrifolia* | N/A | 62.33 | -153.25 | ALA66126 |
| *Rhodiola integrifolia* | N/A | 62.40 | -158.00 | ALA23460 |
| *Rhodiola integrifolia* | N/A | 62.41 | -151.41 | ALA66845 |
| *Rhodiola integrifolia* | N/A | 62.43 | -149.18 | ALA67196 |
| *Rhodiola integrifolia* | N/A | 62.46 | -153.83 | ALA66212 |
| *Rhodiola integrifolia* | N/A | 62.51 | -150.78 | ALA67091 |
| *Rhodiola integrifolia* | N/A | 62.66 | -152.50 | ALA66662 |
| *Rhodiola integrifolia* | N/A | 62.90 | -155.96 | ALA91634 |
| *Rhodiola integrifolia* | N/A | 62.91 | -156.93 | ALA23477 |
| *Rhodiola integrifolia* | N/A | 62.93 | -157.00 | ALA23476 |
| *Rhodiola integrifolia* | N/A | 62.98 | -156.06 | ALA65524 |
| *Rhodiola integrifolia* | N/A | 62.98 | -154.93 | ALA65890 |
| *Rhodiola integrifolia* | N/A | 63.05 | -147.23 | ALA73046 |
| *Rhodiola integrifolia* | N/A | 63.06 | -146.75 | ALA69759 |
| *Rhodiola integrifolia* | N/A | 63.33 | -143.33 | ALA68834 |
| *Rhodiola integrifolia* | N/A | 63.35 | -148.38 | ALA72455 |
| *Rhodiola integrifolia* | N/A | 63.39 | -148.94 | ALA72584 |
| *Rhodiola integrifolia* | N/A | 63.41 | -150.33 | ALA73829 |
| *Rhodiola integrifolia* | N/A | 63.41 | -150.25 | ALA73689 |
| *Rhodiola integrifolia* | N/A | 63.43 | -150.40 | ALA73892 |
| *Rhodiola integrifolia* | N/A | 63.48 | -162.03 | ALA79446 |
| *Rhodiola integrifolia* | N/A | 63.50 | -145.58 | ALA70370 |
| *Rhodiola integrifolia* | N/A | 63.51 | -150.90 | ALA74237 |
| *Rhodiola integrifolia* | N/A | 63.51 | -149.91 | ALA71867 |
| *Rhodiola integrifolia* | N/A | 63.53 | -145.86 | ALA70348 |
| *Rhodiola integrifolia* | N/A | 63.56 | -149.60 | ALA71976 |
| *Rhodiola integrifolia* | N/A | 63.61 | -145.83 | ALA70469 |
| *Rhodiola integrifolia* | N/A | 63.63 | -149.56 | ALA72178 |
| *Rhodiola integrifolia* | N/A | 63.64 | -149.53 | ALA31154 |
| *Rhodiola integrifolia* | N/A | 63.70 | -142.26 | ALA69464 |
| *Rhodiola integrifolia* | N/A | 63.73 | -149.33 | ALA72292 |
| *Rhodiola integrifolia* | N/A | 63.77 | -146.97 | ALA130867 |
| *Rhodiola integrifolia* | N/A | 63.78 | -171.75 | ALA79564 |
| *Rhodiola integrifolia* | N/A | 63.80 | -148.93 | ALA72870 |
| *Rhodiola integrifolia* | N/A | 63.83 | -146.66 | ALA35604 |
| *Rhodiola integrifolia* | N/A | 63.83 | -137.50 | ALA32633 |
| *Rhodiola integrifolia* | N/A | 63.86 | -160.78 | ALA79375 |
| *Rhodiola integrifolia* | N/A | 63.86 | -148.96 | ALA72906 |
| *Rhodiola integrifolia* | N/A | 63.90 | -159.95 | ALA17204 |
| *Rhodiola integrifolia* | N/A | 63.93 | -147.45 | ALA14296 |
| *Rhodiola integrifolia* | N/A | 63.95 | -150.28 | ALA39118 |
| *Rhodiola integrifolia* | N/A | 63.96 | -148.68 | ALA72986 |
| *Rhodiola integrifolia* | N/A | 63.97 | -149.47 | ALA75244 |
| *Rhodiola integrifolia* | N/A | 63.97 | -149.42 | ALA31198 |
| *Rhodiola integrifolia* | N/A | 64.18 | -136.38 | ALA32631 |
| *Rhodiola integrifolia* | N/A | 64.20 | -143.83 | ALA80732 |
| *Rhodiola integrifolia* | N/A | 64.30 | -138.63 | ALA32634 |
| *Rhodiola integrifolia* | N/A | 64.41 | -173.16 | ALA7761 |
| *Rhodiola integrifolia* | N/A | 64.50 | -165.41 | ALA79923 |
| *Rhodiola integrifolia* | N/A | 64.53 | -163.75 | ALA23484 |
| *Rhodiola integrifolia* | N/A | 64.55 | -165.38 | ALA21549 |
| *Rhodiola integrifolia* | N/A | 64.58 | -165.66 | ALA80455 |
| *Rhodiola integrifolia* | N/A | 64.63 | -164.25 | ALA85955 |
| *Rhodiola integrifolia* | N/A | 64.66 | -143.27 | ALA83796 |
| *Rhodiola integrifolia* | N/A | 64.70 | -162.03 | ALA20667 |
| *Rhodiola integrifolia* | N/A | 64.74 | -166.33 | ALA145079 |
| *Rhodiola integrifolia* | N/A | 64.83 | -160.90 | ALA23472 |
| *Rhodiola integrifolia* | N/A | 64.83 | -160.83 | ALA23466 |
| *Rhodiola integrifolia* | N/A | 64.85 | -165.23 | ALA80628 |
| *Rhodiola integrifolia* | N/A | 64.93 | -163.16 | ALA20625 |
| *Rhodiola integrifolia* | N/A | 64.96 | -168.06 | ALA92863 |
| *Rhodiola integrifolia* | N/A | 64.99 | -142.93 | ALA83795 |
| *Rhodiola integrifolia* | N/A | 65.00 | -142.82 | ALA137608 |
| *Rhodiola integrifolia* | N/A | 65.08 | -141.10 | ALA148040 |
| *Rhodiola integrifolia* | N/A | 65.11 | -141.00 | ALA83794 |
| *Rhodiola integrifolia* | N/A | 65.26 | -141.13 | ALA7236 |
| *Rhodiola integrifolia* | N/A | 65.34 | -141.05 | ALA137607 |
| *Rhodiola integrifolia* | N/A | 65.38 | -167.15 | ALA92969 |
| *Rhodiola integrifolia* | N/A | 65.41 | -146.03 | ALA90510 |
| *Rhodiola integrifolia* | N/A | 65.45 | -145.43 | ALA14297 |
| *Rhodiola integrifolia* | N/A | 65.46 | -164.70 | ALA55859 |
| *Rhodiola integrifolia* | N/A | 65.46 | -145.41 | ALA91161 |
| *Rhodiola integrifolia* | N/A | 65.48 | -145.41 | ALA23475 |
| *Rhodiola integrifolia* | N/A | 65.60 | -168.08 | ALA92670 |
| *Rhodiola integrifolia* | N/A | 65.60 | -163.21 | ALA75778 |
| *Rhodiola integrifolia* | N/A | 65.61 | -168.08 | ALA92603 |
| *Rhodiola integrifolia* | N/A | 65.61 | -147.36 | ALA12625 |
| *Rhodiola integrifolia* | N/A | 65.63 | -146.76 | ALA10634 |
| *Rhodiola integrifolia* | N/A | 65.80 | -151.45 | ALA92220 |
| *Rhodiola integrifolia* | N/A | 65.83 | -164.43 | ALA23479 |
| *Rhodiola integrifolia* | N/A | 65.85 | -164.71 | ALA6240 |
| *Rhodiola integrifolia* | N/A | 65.86 | -164.43 | ALA23478 |
| *Rhodiola integrifolia* | N/A | 65.88 | -147.25 | ALA10949 |
| *Rhodiola integrifolia* | N/A | 66.09 | -156.05 | ALA5059 |
| *Rhodiola integrifolia* | N/A | 66.10 | -165.68 | ALA76014 |
| *Rhodiola integrifolia* | N/A | 66.13 | -160.22 | ALA130833 |
| *Rhodiola integrifolia* | N/A | 66.25 | -166.06 | ALA75977 |
| *Rhodiola integrifolia* | N/A | 66.52 | -164.78 | ALA40259 |
| *Rhodiola integrifolia* | N/A | 66.53 | -164.68 | ALA40407 |
| *Rhodiola integrifolia* | N/A | 66.55 | -163.60 | ALA76222 |
| *Rhodiola integrifolia* | N/A | 66.56 | -164.41 | ALA23467 |
| *Rhodiola integrifolia* | N/A | 66.59 | -163.97 | ALA68165 |
| *Rhodiola integrifolia* | N/A | 66.65 | -155.34 | ALA50327 |
| *Rhodiola integrifolia* | N/A | 66.92 | -155.52 | ALA50207 |
| *Rhodiola integrifolia* | N/A | 66.93 | -155.48 | ALA50258 |
| *Rhodiola integrifolia* | N/A | 66.98 | -158.41 | ALA33731 |
| *Rhodiola integrifolia* | N/A | 67.08 | -163.43 | ALA39491 |
| *Rhodiola integrifolia* | N/A | 67.08 | -156.91 | ALA23486 |
| *Rhodiola integrifolia* | N/A | 67.18 | -141.97 | ALA138606 |
| *Rhodiola integrifolia* | N/A | 67.24 | -158.09 | ALA49269 |
| *Rhodiola integrifolia* | N/A | 67.42 | -156.04 | ALA50531 |
| *Rhodiola integrifolia* | N/A | 67.85 | -158.33 | ALA77633 |
| *Rhodiola integrifolia* | N/A | 67.85 | -154.00 | ALA77139 |
| *Rhodiola integrifolia* | N/A | 68.42 | -144.03 | ALA133929 |
| *Rhodiola integrifolia* | N/A | 68.50 | 168.25 | ALA82881 |
| *Rhodiola integrifolia* | Canada: Yukon Territory | 69.31 | -140.48 | V226544 |
| *Rhodiola integrifolia* | N/A | 69.41 | -139.63 | ALA37082 |
| *Rhodiola integrifolia* | Canada: Northwest Territories | 69.44 | -134.45 | V175863 |
| *Rhodiola integrifolia* | N/A | 69.60 | -144.65 | ALA141029 |
| *Rhodiola integrifolia* | N/A | 69.67 | -143.58 | ALA147223 |
| *Rhodiola integrifolia* | N/A | 69.75 | -143.70 | ALA135161 |
| *Rhodiola integrifolia* | N/A | 69.77 | -141.62 | ALA135797 |
| *Rhodiola integrifolia* | N/A | 69.88 | -142.30 | ALA23471 |
| *Rhodiola integrifolia* | N/A | 69.93 | -142.38 | ALA135332 |
| *Rhodiola integrifolia* | N/A | 70.02 | -144.47 | ALA143408 |
| *Rhodiola integrifolia* | N/A | 70.13 | -143.72 | ALA146958 |
| *Rhodiola integrifolia* | N/A | 70.30 | -148.33 | ALA137148 |
| *Rhodiola integrifolia* | N/A | 70.40 | -148.72 | ALA139566 |
| *Rhodiola integrifolia subsp. integrifolia* | USA: CA, Inyo Cty. | 36.48 | -118.23 | RSA509496 |
| *Rhodiola integrifolia subsp. integrifolia* | USA: CA, Tulare Cty. | 36.60 | -118.42 | RSA619665 |
| *Rhodiola integrifolia subsp. integrifolia* | USA: CA, Inyo Cty. | 36.89 | -118.35 | RSA613364 |
| *Rhodiola integrifolia subsp. integrifolia* | USA: CA, Inyo Cty. | 37.24 | -118.68 | POM318836 |
| *Rhodiola integrifolia subsp. integrifolia* | USA: CA, Tuolumne Cty. | 37.91 | -119.24 | RSA717490 |
| *Rhodiola integrifolia subsp. integrifolia* | USA: AK | 57.14 | -154.19 | WTU314009 |
| *Rhodiola integrifolia subsp. integrifolia* | USA: AK | 57.16 | -154.25 | WTU314008 |
| *Rhodiola integrifolia subsp. leedyi* | USA: NY, Yates Cty. | 42.50 | -76.92 | DUL519737 |
| *Rhodiola integrifolia subsp. leedyi* | USA: MN, Fillmore Cty. | 43.73 | -92.34 | DUL787290 |
| *Rhodiola integrifolia subsp. leedyi* | USA: MN, Olmstead Cty. | 43.88 | -92.40 | DUL520352 |
| *Rhodiola integrifolia subsp. leedyi* | USA: MN, Olmstead Cty. | 44.10 | -92.13 | DUL456703 |
| *Rhodiola rosea* | N/A | 62.48 | 171.86 | ALA9147 |
| *Rhodiola rosea* | N/A | 64.73 | 177.50 | ALA8198 |
| *Rhodiola rosea* | N/A | 66.33 | -179.03 | ALA82840 |
| *Sedum integrifolium* | USA: NM, San Miguel Cty. | 35.69 | -105.61 | RM344350 |
| *Sedum integrifolium* | USA: NM, San Miguel Cty. | 35.74 | -105.42 | RM348281 |
| *Sedum integrifolium* | USA: NM, San Miguel Cty. | 35.74 | -105.41 | RM348313 |
| *Sedum integrifolium* | USA: NM, Santa Fe Cty. | 35.79 | -105.76 | RM808680 |
| *Sedum integrifolium* | USA: NM, San Miguel Cty. | 35.83 | -105.62 | RM352495 |
| *Sedum integrifolium* | USA: NM, Santa Fe Cty. | 35.84 | -105.76 | RM350667 |
| *Sedum integrifolium* | USA: NM, San Miguel Cty. | 35.87 | -105.56 | RM345284 |
| *Sedum integrifolium* | USA: NM, San Miguel Cty. | 35.87 | -105.52 | RM345219 |
| *Sedum integrifolium* | USA: NM, Mora Cty. | 35.91 | -105.65 | RM808682 |
| *Sedum integrifolium* | USA: NM, Mora Cty. | 35.94 | -105.70 | USFS462878 |
| *Sedum integrifolium* | USA: NM, Mora Cty. | 35.94 | -105.65 | RM808679 |
| *Sedum integrifolium* | USA: NM, Taos Cty. | 36.78 | -105.49 | RM621779 |
| *Sedum integrifolium* | USA: NM, Colfax Cty. | 36.83 | -105.22 | RM737944 |
| *Sedum integrifolium* | USA: CO, Conejos Cty. | 37.30 | -106.54 | RM164771 |
| *Sedum integrifolium* | USA: CO, Rio Grande Cty. | 37.46 | -106.41 | RM165247 |
| *Sedum integrifolium* | USA: CO, Rio Grande Cty. | 37.47 | -106.56 | RM621382 |
| *Sedum integrifolium* | USA: CO, Montezuma Cty. | 37.48 | -108.11 | USFS462941 |
| *Sedum integrifolium* | USA: CO, Rio Grande Cty. | 37.49 | -106.67 | RM795956 |
| *Sedum integrifolium* | USA: CO, Rio Grande Cty. | 37.49 | -106.69 | RM795957 |
| *Sedum integrifolium* | USA: CO, Rio Grande Cty. | 37.58 | -106.56 | RM795962 |
| *Sedum integrifolium* | USA: CO, Mineral Cty. | 37.61 | -106.85 | RM795961 |
| *Sedum integrifolium* | USA: CO, La Plata Cty. | 37.62 | -107.61 | RM166920 |
| *Sedum integrifolium* | USA: CO, Mineral Cty. | 37.69 | -107.12 | RM795954 |
| *Sedum integrifolium* | USA: CO, Hinsdale Cty. | 37.71 | -107.53 | RM165967 |
| *Sedum integrifolium* | USA: CO, Hinsdale Cty. | 37.71 | -107.35 | RM795953 |
| *Sedum integrifolium* | USA: CO, Rio Grande Cty. | 37.78 | -106.66 | RM795958 |
| *Sedum integrifolium* | USA: CO, San Juan Cty. | 37.78 | -107.53 | RM165469 |
| *Sedum integrifolium* | USA: CO, Hinsdale Cty. | 37.79 | -107.36 | RM795959 |
| *Sedum integrifolium* | USA: CO, San Juan Cty. | 37.80 | -107.55 | RM795955 |
| *Sedum integrifolium* | USA: CO, Hinsdale Cty. | 37.81 | -107.34 | RM165799 |
| *Sedum integrifolium* | USA: CO, Rio Grande Cty. | 37.83 | -106.69 | RM795960 |
| *Sedum integrifolium* | USA: CO, Hinsdale Cty. | 37.85 | -107.37 | RM164517 |
| *Sedum integrifolium* | USA: CO, Hinsdale Cty. | 37.87 | -107.47 | RM165889 |
| *Sedum integrifolium* | USA: CO, Pueblo Cty. | 37.89 | -105.03 | RM733715 |
| *Sedum integrifolium* | USA: CO, Mineral Cty. | 37.90 | -106.78 | RM165568 |
| *Sedum integrifolium* | USA: CO, Mineral Cty. | 37.92 | -106.85 | RM164313 |
| *Sedum integrifolium* | USA: CO, Hinsdale Cty. | 37.92 | -107.48 | RM749083 |
| *Sedum integrifolium* | USA: CO, San Juan Cty. | 37.93 | -107.61 | RM733717 |
| *Sedum integrifolium* | USA: CO, Hinsdale Cty. | 37.96 | -107.04 | RM749082 |
| *Sedum integrifolium* | USA: CO, Saguache Cty. | 38.01 | -106.91 | RM749081 |
| *Sedum integrifolium* | USA: CO, Hinsdale Cty. | 38.07 | -107.46 | RM491795 |
| *Sedum integrifolium* | USA: CO, Chaffee Cty. | 38.62 | -106.27 | RM203249 |
| *Sedum integrifolium* | USA: CO, Chaffee Cty. | 38.79 | -106.38 | RM201758 |
| *Sedum integrifolium* | USA: CO, Chaffee Cty. | 38.83 | -106.39 | RM201806 |
| *Sedum integrifolium* | USA: CO, Chaffee Cty. | 38.91 | -106.30 | RM202057 |
| *Sedum integrifolium* | USA: CO, Chaffee Cty. | 38.95 | -106.38 | RM209240 |
| *Sedum integrifolium* | USA: CO, Chaffee Cty. | 38.95 | -106.43 | RM202693 |
| *Sedum integrifolium* | USA: CO, Chaffee Cty. | 38.99 | -106.16 | RM207888 |
| *Sedum integrifolium* | USA: CO, Chaffee Cty. | 39.02 | -106.56 | RM208717 |
| *Sedum integrifolium* | USA: CO, Chaffee Cty. | 39.02 | -106.41 | RM204272 |
| *Sedum integrifolium* | USA: CO, Chaffee Cty. | 39.04 | -106.49 | RM204125 |
| *Sedum integrifolium* | USA: CO, Pitkin Cty. | 39.05 | -106.60 | RM208861 |
| *Sedum integrifolium* | USA: CO, Pitkin Cty. | 39.08 | -106.60 | RM208765 |
| *Sedum integrifolium* | USA: CO, Lake Cty. | 39.11 | -106.45 | RM621381 |
| *Sedum integrifolium* | USA: CO, Lake Cty. | 39.11 | -106.56 | RM201173 |
| *Sedum integrifolium* | USA: CO, Lake Cty. | 39.12 | -106.41 | RM208894 |
| *Sedum integrifolium* | USA: CO, Lake Cty. | 39.12 | -106.19 | USFS462947 |
| *Sedum integrifolium* | USA: CO, Pitkin Cty. | 39.17 | -106.58 | RM204491 |
| *Sedum integrifolium* | USA: CO, Lake Cty. | 39.17 | -106.19 | RM205964 |
| *Sedum integrifolium* | USA: CO, Lake Cty. | 39.18 | -106.47 | RM202956 |
| *Sedum integrifolium* | USA: CO, Lake Cty. | 39.20 | -106.45 | RM202882 |
| *Sedum integrifolium* | USA: CO, Park Cty. | 39.20 | -106.17 | RM202442 |
| *Sedum integrifolium* | USA: CO, Lake Cty. | 39.21 | -106.19 | RM203038 |
| *Sedum integrifolium* | USA: CO, Lake Cty. | 39.22 | -106.19 | RM203073 |
| *Sedum integrifolium* | USA: CO, Park Cty. | 39.27 | -106.14 | RM397702 |
| *Sedum integrifolium* | USA: CO, Lake Cty. | 39.27 | -106.47 | RM201641 |
| *Sedum integrifolium* | USA: CO, Lake Cty. | 39.28 | -106.19 | RM201514 |
| *Sedum integrifolium* | USA: CO, Park Cty. | 39.34 | -106.14 | RM209640 |
| *Sedum integrifolium* | USA: CO, Pitkin Cty. | 39.35 | -106.60 | USFS462944 |
| *Sedum integrifolium* | USA: CO, Eagle Cty. | 39.37 | -106.54 | RM201281 |
| *Sedum integrifolium* | USA: CO, Summit Cty. | 39.38 | -106.10 | RM345165 |
| *Sedum integrifolium* | USA: CO, Eagle Cty. | 39.38 | -106.49 | RM202270 |
| *Sedum integrifolium* | USA: CO, Eagle Cty. | 39.40 | -106.49 | RM202214 |
| *Sedum integrifolium* | USA: CO, Eagle Cty. | 39.44 | -106.47 | RM204060 |
| *Sedum integrifolium* | USA: CO, Summit Cty. | 39.46 | -105.98 | RM30756 |
| *Sedum integrifolium* | USA: CO, Clear Creek Cty. | 39.58 | -105.64 | RM733712 |
| *Sedum integrifolium* | USA: CO, Grand Cty. | 39.59 | -105.69 | RM176053 |
| *Sedum integrifolium* | USA: CO, Clear Creek Cty. | 39.60 | -105.63 | USFS462867 |
| *Sedum integrifolium* | USA: CO, Clear Creek Cty. | 39.60 | -105.75 | RM172575 |
| *Sedum integrifolium* | USA: CO, Clear Creek Cty. | 39.61 | -105.63 | RM176904 |
| *Sedum integrifolium* | USA: CO, Clear Creek Cty. | 39.64 | -105.82 | RM172022 |
| *Sedum integrifolium* | USA: CO, Clear Creek Cty. | 39.65 | -105.80 | RM170714 |
| *Sedum integrifolium* | USA: CO, Clear Creek Cty. | 39.66 | -105.79 | RM733714 |
| *Sedum integrifolium* | USA: CO, Clear Creek Cty. | 39.68 | -105.91 | RM176162 |
| *Sedum integrifolium* | USA: CO, Clear Creek Cty. | 39.70 | -105.85 | RM181306 |
| *Sedum integrifolium* | USA: CO, Clear Creek Cty. | 39.74 | -105.75 | RM176284 |
| *Sedum integrifolium* | USA: CO, Clear Creek Cty. | 39.74 | -105.85 | RM180159 |
| *Sedum integrifolium* | USA: CO, Grand Cty. | 39.78 | -105.89 | RM170607 |
| *Sedum integrifolium* | USA: CO, Grand Cty. | 39.80 | -105.75 | RM172957 |
| *Sedum integrifolium* | USA: CO, Clear Creek Cty. | 39.85 | -105.67 | RM304402 |
| *Sedum integrifolium* | USA: CO, Grand Cty. | 39.88 | -105.94 | RM170481 |
| *Sedum integrifolium* | USA: CO, Grand Cty. | 39.89 | -105.70 | RM170153 |
| *Sedum integrifolium* | USA: CO, Grand Cty. | 39.92 | -105.68 | RM170225 |
| *Sedum integrifolium* | USA: CO, Grand Cty. | 39.97 | -105.94 | RM179561 |
| *Sedum integrifolium* | USA: CO, Boulder Cty. | 40.01 | -105.58 | RM171652 |
| *Sedum integrifolium* | USA: CO, Grand Cty. | 40.08 | -105.64 | RM171749 |
| *Sedum integrifolium* | USA: CO, Grand Cty. | 40.13 | -105.67 | RM177413 |
| *Sedum integrifolium* | USA: CO, Grand Cty. | 40.33 | -106.13 | RM758425 |
| *Sedum integrifolium* | USA: CO, Grand Cty. | 40.33 | -106.13 | RM170071 |
| *Sedum integrifolium* | USA: CO, Jackson Cty. | 40.37 | -105.96 | RM758427 |
| *Sedum integrifolium* | USA: CO, Jackson Cty. | 40.49 | -105.89 | RM767223 |
| *Sedum integrifolium* | USA: CO, Jackson Cty. | 40.58 | -105.92 | RM758424 |
| *Sedum integrifolium* | USA: CO, Larimer Cty. | 40.60 | -105.93 | RM767224 |
| *Sedum integrifolium* | USA: CO, Jackson Cty. | 40.60 | -105.92 | RM758426 |
| *Sedum integrifolium* | USA: CO, Larimer Cty. | 40.62 | -105.91 | RM767225 |
| *Sedum integrifolium* | USA: CO, Larimer Cty. | 40.68 | -105.95 | RM767226 |
| *Sedum integrifolium* | USA: SD, Pennington Cty. | 43.87 | -103.53 | RM698922 |
| *Sedum integrifolium* | USA: WY, Park Cty. | 44.99 | -109.43 | RM603292 |
| *Sedum integrifolium* | USA: MT, Madison Cty. | 45.05 | -111.44 | RM783906 |
| *Sedum integrifolium* | USA: MT, Stillwater Cty. | 45.36 | -109.82 | RM780092 |
| *Sedum integrifolium* | USA: WA, Chelan Cty. | 48.52 | -120.81 | WTU374292 |
| *Sedum integrifolium* | Canada: British Columbia | 52.38 | -126.75 | V220068 |
| *Sedum integrifolium subsp. integrifolium* | USA: NM, Lincoln Cty. | 33.33 | -105.67 | RM222222 |
| *Sedum integrifolium subsp. integrifolium* | USA: NM, Otero Cty. | 33.38 | -105.81 | RM286300 |
| *Sedum integrifolium subsp. integrifolium* | USA: NM, Lincoln Cty. | 33.39 | -105.81 | USFS462859 |
| *Sedum integrifolium subsp. integrifolium* | USA: NM, Bernalillo Cty. | 35.21 | -106.45 | RM127993 |
| *Sedum integrifolium subsp. integrifolium* | USA: NM, Taos Cty. | 36.59 | -105.42 | RM263737 |
| *Sedum integrifolium subsp. integrifolium* | USA: NM, Taos Cty. | 36.79 | -105.47 | RM260325 |
| *Sedum integrifolium subsp. integrifolium* | USA: CO, Huerfano Cty. | 37.29 | -105.16 | RM76350 |
| *Sedum integrifolium subsp. integrifolium* | USA: CO, Huerfano Cty. | 37.29 | -105.17 | RM76445 |
| *Sedum integrifolium subsp. integrifolium* | USA: CO, Huerfano Cty. | 37.33 | -105.24 | RM72738 |
| *Sedum integrifolium subsp. integrifolium* | USA: CO, Las Animas Cty. | 37.35 | -105.02 | RM69400 |
| *Sedum integrifolium subsp. integrifolium* | USA: CO, Las Animas Cty. | 37.36 | -105.00 | RM69453 |
| *Sedum integrifolium subsp. integrifolium* | USA: CO, Huerfano Cty. | 37.37 | -104.98 | RM77128 |
| *Sedum integrifolium subsp. integrifolium* | USA: CO, Huerfano Cty. | 37.39 | -104.91 | RM70025 |
| *Sedum integrifolium subsp. integrifolium* | USA: CO, Montezuma Cty. | 37.45 | -108.08 | RM707936 |
| *Sedum integrifolium subsp. integrifolium* | USA: CO, Montezuma Cty. | 37.45 | -108.06 | RM712309 |
| *Sedum integrifolium subsp. integrifolium* | USA: CO, Alamosa Cty. | 37.57 | -105.50 | RM70409 |
| *Sedum integrifolium subsp. integrifolium* | USA: CO, Huerfano Cty. | 37.59 | -105.46 | RM78603 |
| *Sedum integrifolium subsp. integrifolium* | USA: CO, Huerfano Cty. | 37.59 | -105.49 | RM73044 |
| *Sedum integrifolium subsp. integrifolium* | USA: CO, Alamosa Cty. | 37.61 | -105.51 | RM82907 |
| *Sedum integrifolium subsp. integrifolium* | USA: CO, Dolores Cty. | 37.68 | -107.99 | RM712306 |
| *Sedum integrifolium subsp. integrifolium* | USA: CO, Dolores Cty. | 37.70 | -107.95 | RM707957 |
| *Sedum integrifolium subsp. integrifolium* | USA: CO, Dolores Cty. | 37.70 | -107.93 | RM707959 |
| *Sedum integrifolium subsp. integrifolium* | USA: CO, Dolores Cty. | 37.72 | -107.90 | RM707937 |
| *Sedum integrifolium subsp. integrifolium* | USA: CO, Dolores Cty. | 37.73 | -107.88 | RM712305 |
| *Sedum integrifolium subsp. integrifolium* | USA: CO, Dolores Cty. | 37.74 | -108.06 | RM712304 |
| *Sedum integrifolium subsp. integrifolium* | USA: CO, Dolores Cty. | 37.78 | -107.98 | RM712300 |
| *Sedum integrifolium subsp. integrifolium* | USA: CO, Dolores Cty. | 37.80 | -108.20 | RM712303 |
| *Sedum integrifolium subsp. integrifolium* | USA: CO, Dolores Cty. | 37.81 | -107.96 | RM707958 |
| *Sedum integrifolium subsp. integrifolium* | USA: CO, San Miguel Cty. | 37.83 | -108.20 | RM283860 |
| *Sedum integrifolium subsp. integrifolium* | USA: CO, Dolores Cty. | 37.83 | -108.12 | RM712301 |
| *Sedum integrifolium subsp. integrifolium* | USA: CO, San Miguel Cty. | 37.83 | -107.87 | RM286120 |
| *Sedum integrifolium subsp. integrifolium* | USA: CO, Dolores Cty. | 37.84 | -108.10 | RM712302 |
| *Sedum integrifolium subsp. integrifolium* | USA: CO, San Miguel Cty. | 37.84 | -107.94 | RM281003 |
| *Sedum integrifolium subsp. integrifolium* | USA: CO, San Miguel Cty. | 37.86 | -108.05 | RM280982 |
| *Sedum integrifolium subsp. integrifolium* | USA: CO, San Miguel Cty. | 37.89 | -107.99 | RM281166 |
| *Sedum integrifolium subsp. integrifolium* | USA: CO, Pueblo Cty. | 37.89 | -105.02 | RM72425 |
| *Sedum integrifolium subsp. integrifolium* | USA: CO, Huerfano Cty. | 37.89 | -105.00 | RM76028 |
| *Sedum integrifolium subsp. integrifolium* | USA: CO, San Miguel Cty. | 37.90 | -108.23 | RM285254 |
| *Sedum integrifolium subsp. integrifolium* | USA: CO, Pueblo Cty. | 37.90 | -105.04 | RM75990 |
| *Sedum integrifolium subsp. integrifolium* | USA: CO, Hinsdale Cty. | 37.92 | -107.51 | RM300100 |
| *Sedum integrifolium subsp. integrifolium* | USA: CO, Saguache Cty. | 37.93 | -105.53 | RM82342 |
| *Sedum integrifolium subsp. integrifolium* | USA: CO, Hinsdale Cty. | 37.94 | -107.42 | RM301530 |
| *Sedum integrifolium subsp. integrifolium* | USA: CO, Custer Cty. | 37.96 | -105.55 | RM70647 |
| *Sedum integrifolium subsp. integrifolium* | USA: CO, Custer Cty. | 37.96 | -105.51 | RM70191 |
| *Sedum integrifolium subsp. integrifolium* | USA: CO, Custer Cty. | 37.98 | -105.55 | RM70730 |
| *Sedum integrifolium subsp. integrifolium* | USA: CO, Saguache Cty. | 37.99 | -105.65 | RM83203 |
| *Sedum integrifolium subsp. integrifolium* | USA: CO, Saguache Cty. | 37.99 | -105.64 | RM83248 |
| *Sedum integrifolium subsp. integrifolium* | USA: CO, Saguache Cty. | 37.99 | -105.63 | RM83151 |
| *Sedum integrifolium subsp. integrifolium* | USA: CO, San Miguel Cty. | 38.02 | -107.94 | RM286948 |
| *Sedum integrifolium subsp. integrifolium* | USA: CO, Custer Cty. | 38.05 | -105.61 | RM82677 |
| *Sedum integrifolium subsp. integrifolium* | USA: CO, Hinsdale Cty. | 38.06 | -107.42 | RM300760 |
| *Sedum integrifolium subsp. integrifolium* | USA: CO, Custer Cty. | 38.06 | -105.58 | RM82806 |
| *Sedum integrifolium subsp. integrifolium* | USA: CO, Custer Cty. | 38.09 | -105.65 | RM68674 |
| *Sedum integrifolium subsp. integrifolium* | USA: CO, Gunnison Cty. | 38.21 | -107.48 | RM298967 |
| *Sedum integrifolium subsp. integrifolium* | USA: CO, Custer Cty. | 38.22 | -105.70 | RM69206 |
| *Sedum integrifolium subsp. integrifolium* | USA: CO, Saguache Cty. | 38.29 | -106.09 | RM77949 |
| *Sedum integrifolium subsp. integrifolium* | USA: CO, Saguache Cty. | 38.33 | -106.21 | RM69294 |
| *Sedum integrifolium subsp. integrifolium* | USA: CO, Chaffee Cty. | 38.35 | -106.15 | RM82552 |
| *Sedum integrifolium subsp. integrifolium* | USA: CO, Fremont Cty. | 38.35 | -105.87 | RM69703 |
| *Sedum integrifolium subsp. integrifolium* | USA: CO, Gunnison Cty. | 38.48 | -107.49 | RM720778 |
| *Sedum integrifolium subsp. integrifolium* | USA: CO, Gunnison Cty. | 38.64 | -106.43 | RM720759 |
| *Sedum integrifolium subsp. integrifolium* | USA: CO, Gunnison Cty. | 38.65 | -106.63 | RM235950 |
| *Sedum integrifolium subsp. integrifolium* | USA: CO, Gunnison Cty. | 38.67 | -107.19 | RM236775 |
| *Sedum integrifolium subsp. integrifolium* | USA: CO, Gunnison Cty. | 38.68 | -106.61 | RM236022 |
| *Sedum integrifolium subsp. integrifolium* | USA: CO, Gunnison Cty. | 38.70 | -107.16 | RM242319 |
| *Sedum integrifolium subsp. integrifolium* | USA: CO, Gunnison Cty. | 38.70 | -106.53 | RM720763 |
| *Sedum integrifolium subsp. integrifolium* | USA: CO, Gunnison Cty. | 38.70 | -107.19 | RM236923 |
| *Sedum integrifolium subsp. integrifolium* | USA: CO, Gunnison Cty. | 38.73 | -107.17 | RM242093 |
| *Sedum integrifolium subsp. integrifolium* | USA: CO, Teller Cty. | 38.77 | -105.03 | RM96554 |
| *Sedum integrifolium subsp. integrifolium* | USA: CO, Gunnison Cty. | 38.82 | -106.99 | RM719351 |
| *Sedum integrifolium subsp. integrifolium* | USA: CO, Gunnison Cty. | 38.82 | -106.67 | RM240495 |
| *Sedum integrifolium subsp. integrifolium* | USA: CO, Gunnison Cty. | 38.83 | -106.66 | RM240555 |
| *Sedum integrifolium subsp. integrifolium* | USA: CO, Teller Cty. | 38.88 | -105.12 | RM95726 |
| *Sedum integrifolium subsp. integrifolium* | USA: CO, Gunnison Cty. | 38.91 | -106.84 | RM720777 |
| *Sedum integrifolium subsp. integrifolium* | USA: CO, Gunnison Cty. | 38.91 | -106.69 | RM240725 |
| *Sedum integrifolium subsp. integrifolium* | USA: CO, Gunnison Cty. | 38.92 | -106.77 | RM720762 |
| *Sedum integrifolium subsp. integrifolium* | USA: CO, Gunnison Cty. | 38.93 | -106.71 | RM240785 |
| *Sedum integrifolium subsp. integrifolium* | USA: CO, Gunnison Cty. | 38.95 | -107.10 | RM236545 |
| *Sedum integrifolium subsp. integrifolium* | USA: CO, Gunnison Cty. | 38.95 | -106.88 | RM720765 |
| *Sedum integrifolium subsp. integrifolium* | USA: CO, Gunnison Cty. | 38.95 | -106.58 | RM720764 |
| *Sedum integrifolium subsp. integrifolium* | USA: CO, Gunnison Cty. | 38.98 | -106.99 | RM720760 |
| *Sedum integrifolium subsp. integrifolium* | USA: CO, Gunnison Cty. | 38.99 | -107.05 | RM720761 |
| *Sedum integrifolium subsp. integrifolium* | USA: CO, Gunnison Cty. | 38.99 | -106.93 | RM232895 |
| *Sedum integrifolium subsp. integrifolium* | USA: CO, Park Cty. | 38.99 | -106.08 | RM95859 |
| *Sedum integrifolium subsp. integrifolium* | USA: CO, Gunnison Cty. | 39.01 | -106.64 | RM241246 |
| *Sedum integrifolium subsp. integrifolium* | USA: CO, Park Cty. | 39.12 | -106.19 | RM97030 |
| *Sedum integrifolium subsp. integrifolium* | USA: CO, Park Cty. | 39.14 | -106.17 | RM100010 |
| *Sedum integrifolium subsp. integrifolium* | USA: CO, Park Cty. | 39.35 | -106.04 | RM100659 |
| *Sedum integrifolium subsp. integrifolium* | USA: CO, Park Cty. | 39.41 | -105.97 | RM96331 |
| *Sedum integrifolium subsp. integrifolium* | USA: CO, Garfield Cty. | 40.08 | -107.11 | RM684086 |
| *Sedum integrifolium subsp. integrifolium* | USA: CO, Jackson Cty. | 40.47 | -106.66 | RM571310 |
| *Sedum integrifolium subsp. integrifolium* | USA: WY, Albany Cty. | 41.39 | -106.26 | RM590301 |
| *Sedum integrifolium subsp. integrifolium* | USA: WY, Fremont Cty. | 42.61 | -108.97 | USFS291510 |
| *Sedum integrifolium subsp. integrifolium* | USA: WY, Fremont Cty. | 42.67 | -109.11 | USFS291695 |
| *Sedum integrifolium subsp. integrifolium* | USA: WY, Sublette Cty. | 42.70 | -109.22 | RM587869 |
| *Sedum integrifolium subsp. integrifolium* | USA: WY, Sublette Cty. | 42.73 | -109.26 | RM587866 |
| *Sedum integrifolium subsp. integrifolium* | USA: WY, Sublette Cty. | 42.74 | -109.16 | RM587868 |
| *Sedum integrifolium subsp. integrifolium* | USA: WY, Fremont Cty. | 42.77 | -109.14 | USFS296366 |
| *Sedum integrifolium subsp. integrifolium* | USA: WY, Fremont Cty. | 42.78 | -109.21 | USFS296286 |
| *Sedum integrifolium subsp. integrifolium* | USA: WY, Sublette Cty. | 42.79 | -109.28 | RM587867 |
| *Sedum integrifolium subsp. integrifolium* | USA: WY, Fremont Cty. | 42.81 | -109.12 | USFS290381 |
| *Sedum integrifolium subsp. integrifolium* | USA: WY, Fremont Cty. | 42.82 | -109.19 | USFS296814 |
| *Sedum integrifolium subsp. integrifolium* | USA: WY, Sublette Cty. | 42.83 | -109.34 | RM627310 |
| *Sedum integrifolium subsp. integrifolium* | USA: WY, Fremont Cty. | 42.85 | -109.29 | USFS296673 |
| *Sedum integrifolium subsp. integrifolium* | USA: WY, Sublette Cty. | 42.89 | -109.38 | RM628538 |
| *Sedum integrifolium subsp. integrifolium* | USA: WY, Sublette Cty. | 42.96 | -109.52 | RM626864 |
| *Sedum integrifolium subsp. integrifolium* | USA: WY, Fremont Cty. | 43.02 | -109.55 | USFS297107 |
| *Sedum integrifolium subsp. integrifolium* | USA: WY, Sublette Cty. | 43.05 | -109.67 | RM620395 |
| *Sedum integrifolium subsp. integrifolium* | USA: WY, Sublette Cty. | 43.05 | -109.65 | RM631696 |
| *Sedum integrifolium subsp. integrifolium* | USA: WY, Fremont Cty. | 43.09 | -109.48 | USFS297436 |
| *Sedum integrifolium subsp. integrifolium* | USA: WY, Fremont Cty. | 43.13 | -109.57 | USFS297665 |
| *Sedum integrifolium subsp. integrifolium* | USA: WY, Sublette Cty. | 43.16 | -109.72 | RM632533 |
| *Sedum integrifolium subsp. integrifolium* | USA: WY, Sublette Cty. | 43.16 | -109.88 | RM631514 |
| *Sedum integrifolium subsp. integrifolium* | USA: WY, Fremont Cty. | 43.17 | -109.61 | USFS292199 |
| *Sedum integrifolium subsp. integrifolium* | USA: WY, Sublette Cty. | 43.20 | -109.82 | RM634162 |
| *Sedum integrifolium subsp. integrifolium* | USA: WY, Fremont Cty. | 43.21 | -109.51 | USFS292039 |
| *Sedum integrifolium subsp. integrifolium* | USA: WY, Fremont Cty. | 43.22 | -109.54 | USFS292089 |
| *Sedum integrifolium subsp. integrifolium* | USA: WY, Sublette Cty. | 43.23 | -109.70 | RM628760 |
| *Sedum integrifolium subsp. integrifolium* | USA: WY, Sublette Cty. | 43.29 | -109.76 | RM632968 |
| *Sedum integrifolium subsp. integrifolium* | USA: WY, Sublette Cty. | 43.32 | -109.74 | RM633836 |
| *Sedum integrifolium subsp. integrifolium* | USA: WY, Fremont Cty. | 43.34 | -109.59 | USFS297774 |
| *Sedum integrifolium subsp. integrifolium* | USA: WY, Fremont Cty. | 43.36 | -109.59 | RM103687 |
| *Sedum integrifolium subsp. integrifolium* | USA: WY, Fremont Cty. | 43.37 | -109.73 | USFS291191 |
| *Sedum integrifolium subsp. integrifolium* | USA: WY, Fremont Cty. | 43.37 | -109.71 | USFS291185 |
| *Sedum integrifolium subsp. integrifolium* | USA: WY, Fremont Cty. | 43.38 | -109.57 | RM102452 |
| *Sedum integrifolium subsp. integrifolium* | USA: WY, Fremont Cty. | 43.38 | -109.56 | RM821240 |
| *Sedum integrifolium subsp. integrifolium* | USA: WY, Fremont Cty. | 43.41 | -109.65 | RM105853 |
| *Sedum integrifolium subsp. integrifolium* | USA: WY, Fremont Cty. | 43.41 | -109.58 | USFS294797 |
| *Sedum integrifolium subsp. integrifolium* | USA: WY, Fremont Cty. | 43.44 | -109.62 | RM710507 |
| *Sedum integrifolium subsp. integrifolium* | USA: WY, Fremont Cty. | 43.44 | -109.64 | USFS290966 |
| *Sedum integrifolium subsp. integrifolium* | USA: WY, Sublette Cty. | 43.44 | -109.78 | RM631794 |
| *Sedum integrifolium subsp. integrifolium* | USA: WY, Fremont Cty. | 43.62 | -109.98 | RM107576 |
| *Sedum integrifolium subsp. Integrifolium* | USA: WY, Teton Cty. | 43.75 | -110.77 | RM838381 |
| *Sedum integrifolium subsp. integrifolium* | USA: WY, Hot Springs Cty. | 43.75 | -109.20 | RM602045 |
| *Sedum integrifolium subsp. Integrifolium* | USA: WY, Teton Cty. | 43.78 | -110.78 | RM838382 |
| *Sedum integrifolium subsp. integrifolium* | USA: WY, Hot Springs Cty. | 43.84 | -109.06 | RM605515 |
| *Sedum integrifolium subsp. integrifolium* | USA: WY, Park Cty. | 43.84 | -109.28 | RM613537 |
| *Sedum integrifolium subsp. integrifolium* | USA: WY, Park Cty. | 43.87 | -109.36 | RM606365 |
| *Sedum integrifolium subsp. integrifolium* | USA: WY, Park Cty. | 43.90 | -109.20 | RM605677 |
| *Sedum integrifolium subsp. integrifolium* | USA: WY, Park Cty. | 43.91 | -109.31 | RM613737 |
| *Sedum integrifolium subsp. integrifolium* | USA: WY, Park Cty. | 44.01 | -109.25 | RM612219 |
| *Sedum integrifolium subsp. integrifolium* | USA: WY, Park Cty. | 44.01 | -109.22 | RM605905 |
| *Sedum integrifolium subsp. integrifolium* | USA: WY, Johnson Cty. | 44.13 | -107.08 | RM323935 |
| *Sedum integrifolium subsp. integrifolium* | USA: WY, Park Cty. | 44.14 | -109.41 | RM601950 |
| *Sedum integrifolium subsp. integrifolium* | USA: WY, Park Cty. | 44.19 | -109.41 | RM603238 |
| *Sedum integrifolium subsp. integrifolium* | USA: WY, Park Cty. | 44.19 | -109.28 | RM606088 |
| *Sedum integrifolium subsp. integrifolium* | USA: WY, Park Cty. | 44.25 | -109.20 | RM603162 |
| *Sedum integrifolium subsp. integrifolium* | USA: WY, Park Cty. | 44.27 | -109.91 | RM625079 |
| *Sedum integrifolium subsp. integrifolium* | USA: WY, Park Cty. | 44.29 | -109.91 | RM732757 |
| *Sedum integrifolium subsp. integrifolium* | USA: WY, Park Cty. | 44.29 | -109.22 | RM731607 |
| *Sedum integrifolium subsp. integrifolium* | USA: WY, Park Cty. | 44.29 | -109.21 | RM612564 |
| *Sedum integrifolium subsp. integrifolium* | USA: WY, Park Cty. | 44.33 | -109.56 | RM103412 |
| *Sedum integrifolium subsp. integrifolium* | USA: WY, Park Cty. | 44.37 | -109.56 | RM371939 |
| *Sedum integrifolium subsp. integrifolium* | USA: WY, Johnson Cty. | 44.42 | -107.13 | RM608028 |
| *Sedum integrifolium subsp. integrifolium* | USA: WY, Park Cty. | 44.42 | -109.50 | RM753040 |
| *Sedum integrifolium subsp. integrifolium* | USA: WY, Park Cty. | 44.43 | -109.48 | RM732381 |
| *Sedum integrifolium subsp. integrifolium* | USA: WY, Johnson Cty. | 44.55 | -107.33 | RM618433 |
| *Sedum integrifolium subsp. integrifolium* | USA: WY, Park Cty. | 44.60 | -109.51 | RM616900 |
| *Sedum integrifolium subsp. integrifolium* | USA: WY, Park Cty. | 44.60 | -109.49 | RM751529 |
| *Sedum integrifolium subsp. integrifolium* | USA: ID, Fremont Cty. | 44.70 | -111.39 | RM692805 |
| *Sedum integrifolium subsp. integrifolium* | USA: WY, Park Cty. | 44.70 | -109.72 | RM569063 |
| *Sedum integrifolium subsp. integrifolium* | USA: ID, Fremont Cty. | 44.74 | -111.37 | RM692806 |
| *Sedum integrifolium subsp. Integrifolium* | USA: ID, Fremont Cty. | 44.75 | -111.40 | RM740359 |
| *Sedum integrifolium subsp. integrifolium* | USA: WY, Park Cty. | 44.85 | -109.80 | RM618059 |
| *Sedum integrifolium subsp. integrifolium* | USA: WY, Park Cty. | 44.94 | -109.94 | RM568734 |
| *Sedum integrifolium subsp. integrifolium* | USA: WY, Park Cty. | 44.98 | -109.92 | RM615218 |
| *Sedum integrifolium subsp. integrifolium* | USA: MT, Carbon Cty. | 45.01 | -109.41 | RM139591 |
| *Sedum integrifolium subsp. integrifolium* | USA: MT, Carbon Cty. | 45.15 | -109.68 | RM780702 |
| *Sedum integrifolium subsp. integrifolium* | USA: MT, Carbon Cty. | 45.15 | -109.56 | RM139404 |
| *Sedum integrifolium subsp. integrifolium* | USA: MT, Madison Cty. | 45.17 | -111.49 | RM740006 |
| *Sedum integrifolium subsp. integrifolium* | USA: MT, Stillwater Cty. | 45.22 | -109.74 | RM787692 |
| *Sedum integrifolium subsp. integrifolium* | USA: MT, Park Cty. | 45.30 | -110.65 | RM821557 |
| *Sedum integrifolium subsp. integrifolium* | USA: MT, Gallatin Cty. | 45.44 | -111.01 | RM822233 |
| *Sedum integrifolium subsp. integrifolium* | USA: MT, Park Cty. | 45.50 | -110.46 | RM139666 |
| *Sedum integrifolium subsp. integrifolium* | USA: ID, Fremont Cty. | 47.17 | -106.29 | RM142201 |
| *Sedum integrifolium subsp. integrifolium* | Canada: British Columbia | 58.00 | -129.00 | V197971 |
| *Sedum integrifolium subsp. integrifolium* | Canada: British Columbia | 58.00 | -128.00 | V188142 |
| *Sedum integrifolium subsp. integrifolium* | Canada: British Columbia | 49.04 | -121.75 | V216627 |
| *Sedum integrifolium subsp. procerum* | USA: NM, Rio Arriba Cty. | 35.98 | -105.63 | RM265509 |
| *Sedum integrifolium subsp. procerum* | USA: NM, Taos Cty. | 36.04 | -105.55 | RM258965 |
| *Sedum integrifolium subsp. procerum* | USA: NM, Taos Cty. | 36.04 | -105.67 | RM259755 |
| *Sedum integrifolium subsp. procerum* | USA: NM, Taos Cty. | 36.06 | -105.50 | RM264921 |
| *Sedum integrifolium subsp. procerum* | USA: NM, Mora Cty. | 36.25 | -105.34 | RM264702 |
| *Sedum integrifolium subsp. procerum* | USA: NM, Taos Cty. | 36.52 | -105.45 | RM268799 |
| *Sedum integrifolium subsp. procerum* | USA: NM, Colfax Cty. | 36.54 | -105.22 | RM265323 |
| *Sedum integrifolium subsp. procerum* | USA: NM, Taos Cty. | 36.57 | -105.39 | RM271512 |
| *Sedum integrifolium subsp. procerum* | USA: NM, Taos Cty. | 36.57 | -105.44 | RM269159 |
| *Sedum integrifolium subsp. procerum* | USA: NM, Taos Cty. | 36.61 | -105.54 | RM261641 |
| *Sedum integrifolium subsp. procerum* | USA: NM, Taos Cty. | 36.63 | -105.55 | RM265888 |
| *Sedum integrifolium subsp. procerum* | USA: NM, Taos Cty. | 36.64 | -105.44 | RM269713 |
| *Sedum integrifolium subsp. procerum* | USA: NM, Taos Cty. | 36.68 | -105.52 | RM271337 |
| *Sedum integrifolium subsp. procerum* | USA: NM, Taos Cty. | 36.78 | -105.50 | RM270573 |
| *Sedum integrifolium subsp. procerum* | USA: NM, Taos Cty. | 36.78 | -105.36 | RM269346 |
| *Sedum integrifolium subsp. procerum* | USA: NM, Taos Cty. | 36.83 | -105.20 | RM260770 |
| *Sedum integrifolium subsp. procerum* | USA: NM, Colfax Cty. | 36.87 | -105.19 | RM708588 |
| *Sedum integrifolium subsp. procerum* | USA: NM, Taos Cty. | 36.92 | -105.33 | RM705757 |
| *Sedum integrifolium subsp. procerum* | USA: NM, Taos Cty. | 36.96 | -105.24 | RM704493 |
| *Sedum integrifolium subsp. procerum* | USA: NM, Taos Cty. | 36.97 | -105.30 | RM708857 |
| *Sedum integrifolium subsp. procerum* | USA: NM, Taos Cty. | 36.98 | -105.30 | RM709000 |
| *Sedum integrifolium subsp. procerum* | USA: NM, Colfax Cty. | 36.99 | -105.23 | RM705415 |
| *Sedum integrifolium subsp. procerum* | USA: NM, Costilla Cty. | 37.02 | -105.24 | RM704126 |
| *Sedum rosea* | USA: CA, Fresno Cty. | 36.77 | -118.39 | UCR24172 |
| *Sedum rosea* | USA: CA, Modoc Cty. | 41.38 | -120.22 | UCR34756 |
| *Sedum rosea* | USA: OR, Baker Cty. | 45.04 | -117.25 | OSU48344 |
| *Sedum rosea* | USA: OR, Wallowa Cty. | 45.29 | -117.34 | BAKER7318 |
| *Sedum rosea* | USA: WA, Chelan Cty. | 48.11 | -120.74 | WTU26725 |
| *Sedum rosea* | USA: WA, Okanogan Cty. | 48.69 | -119.94 | WTU20904 |
| *Sedum rosea* | USA: WA, Whatcom Cty. | 48.85 | -121.85 | WTU20733 |
| *Sedum rosea* | Canada: British Columbia | 49.00 | -116.00 | V167133 |
| *Sedum rosea* | Canada: Alberta | 49.03 | -113.98 | V133649 |
| *Sedum rosea* | Canada: British Columbia | 50.75 | -118.87 | V183020 |
| *Sedum rosea* | USA: AK | 59.42 | -139.02 | WTU282222 |
| *Sedum rosea* | Canada: British Columbia | 59.58 | -136.48 | WTU281873 |
| *Sedum rosea* | Canada: British Columbia | 59.88 | -136.73 | WTU281836 |
| *Sedum rosea* | Canada: Yukon Territory | 60.00 | -137.00 | V180571 |
| *Sedum rosea* | USA: AK | 60.10 | -149.45 | V60384 |
| *Sedum rosea* | Canada: Yukon Territory | 60.92 | -138.57 | V204950 |
| *Sedum rosea* | Canada: Yukon Territory | 63.93 | -135.22 | V200602 |
| *Sedum rosea subsp. integrifolium* | USA: CA, Inyo Cty. | 36.58 | -118.25 | UCR18952 |
| *Sedum rosea subsp. integrifolium* | USA: CA, Inyo Cty. | 37.21 | -118.62 | SJSU7453 |
| *Sedum rosea subsp. integrifolium* | USA: CA, Siskiyou Cty. | 41.31 | -122.94 | SJSU6535 |
| *Sedum rosea subsp. integrifolium* | USA: OR, Harney Cty. | 42.01 | -118.67 | OSC35292 |
| *Sedum rosea subsp. integrifolium* | USA: OR, Harney Cty. | 42.64 | -118.59 | OSU333394 |
| *Sedum rosea subsp. integrifolium* | USA: OR, Harney Cty. | 42.66 | -118.59 | BLMB354 |
| *Sedum rosea subsp. integrifolium* | USA: OR, Harney Cty. | 42.67 | -118.65 | OSC163845 |
| *Sedum rosea subsp. integrifolium* | USA: OR, Harney Cty. | 42.68 | -118.59 | BLMB355 |
| *Sedum rosea subsp. integrifolium* | USA: OR, Harney Cty. | 42.69 | -118.57 | OSC89024 |
| *Sedum rosea subsp. integrifolium* | USA: OR, Harney Cty. | 42.72 | -118.57 | OSC88842 |
| *Sedum rosea subsp. integrifolium* | USA: OR, Harney Cty. | 42.73 | -118.57 | OSU55012 |
| *Sedum rosea subsp. integrifolium* | USA: OR, Grant Cty. | 44.28 | -118.68 | OSC186972 |
| *Sedum rosea subsp. integrifolium* | USA: OR, Linn Cty. | 44.29 | -122.87 | OSU12428 |
| *Sedum rosea subsp. integrifolium* | USA: OR, Grant Cty. | 44.31 | -118.71 | OSU193440 |
| *Sedum rosea subsp. integrifolium* | USA: OR, Grant Cty. | 44.31 | -118.69 | OSU66346 |
| *Sedum rosea subsp. integrifolium* | USA: OR, Baker Cty. | 44.82 | -118.11 | WILLU5764 |
| *Sedum rosea subsp. integrifolium* | USA: OR, Baker Cty. | 44.87 | -118.06 | OSU121609 |
| *Sedum rosea subsp. integrifolium* | USA: OR, Baker Cty. | 44.96 | -118.23 | WILLU19848 |
| *Sedum rosea subsp. integrifolium* | USA: OR, Baker Cty. | 44.96 | -118.23 | WILLU5750 |
| *Sedum rosea subsp. integrifolium* | USA: OR, Baker or Union Cty. | 44.99 | -118.08 | OSU361371 |
| *Sedum rosea subsp. integrifolium* | USA: OR, Wallowa Cty. | 45.16 | -117.29 | ORE41723 |
| *Sedum rosea subsp. integrifolium* | USA: OR, Wallowa Cty. | 45.21 | -117.43 | ORE3896 |
| *Sedum rosea subsp. integrifolium* | USA: OR, Wallowa Cty. | 45.23 | -117.27 | WILLU18336 |
| *Sedum rosea subsp. integrifolium* | USA: OR, Wallowa Cty. | 45.23 | -117.28 | ORE2833 |
| *Sedum rosea subsp. integrifolium* | USA: OR, Wallowa Cty. | 45.23 | -117.32 | WILLU25480 |
| *Sedum rosea subsp. integrifolium* | USA: OR, Wallowa Cty. | 45.28 | -117.30 | OSU150127 |
| *Sedum rosea subsp. integrifolium* | USA: OR, Wallowa Cty. | 45.29 | -117.43 | ORE67450 |
| *Sedum rosea subsp. integrifolium* | USA: OR, Wallowa Cty. | 45.30 | -117.30 | OSU361831 |
| *Sedum rosea subsp. integrifolium* | USA: OR, Wallowa Cty. | 45.36 | -117.36 | ORE3001 |
| *Sedum rosea subsp. integrifolium* | Canada: Yukon Territory | 64.18 | -140.35 | WTU290826 |
| *Sedum roseum* | USA: WA, Pierce Cty. | 46.93 | -121.67 | WTU367423 |
| *Sedum roseum* | USA: WA, Chelan Cty. | 47.48 | -120.81 | WTU262807 |
| *Sedum roseum* | USA: WA, Okanogan Cty. | 48.67 | -119.93 | WTU361353 |
| *Sedum roseum* | Canada: British Columbia | 49.00 | -120.00 | V145432 |
| *Sedum roseum* | Canada: British Columbia | 49.18 | -120.08 | V145433 |
| *Sedum roseum* | Canada: British Columbia | 54.00 | -126.00 | V109753 |
| *Sedum roseum* | Canada: British Columbia | 54.17 | -133.05 | V124219 |
| *Sedum roseum* | Canada: British Columbia | 54.18 | -133.00 | V53237 |
| *Sedum roseum* | Canada: British Columbia | 54.33 | -129.33 | V37666 |
| *Sedum roseum* | Canada: British Columbia | 54.52 | -127.23 | V152596 |
| *Sedum roseum* | Canada: British Columbia | 54.80 | -128.68 | V166249 |
| *Sedum roseum* | Canada: British Columbia | 54.82 | -127.27 | V75365 |
| *Sedum roseum* | Canada: British Columbia | 54.92 | -126.92 | V215511 |
| *Sedum roseum* | Canada: British Columbia | 55.00 | -127.00 | V104488 |
| *Sedum roseum* | Canada: British Columbia | 57.00 | -129.00 | V145406 |
| *Sedum roseum* | Canada: British Columbia | 57.63 | -130.55 | V147779 |
| *Sedum roseum* | Canada: British Columbia | 58.00 | -130.00 | V162897 |
| *Sedum roseum* | Canada: British Columbia | 58.50 | -130.33 | V98144 |
| *Sedum roseum* | Canada: Yukon Territory | 63.00 | -130.55 | V212769 |
| *Sedum roseum* | Canada: Yukon Territory | 64.62 | -139.40 | V212772 |
| *Sedum roseum subsp. integrifolium* | USA: CA, Tulare Cty. | 36.41 | -118.54 | UC1558294 |
| *Sedum roseum subsp. integrifolium* | USA: CA, Tulare Cty. | 36.42 | -118.61 | UC64683 |
| *Sedum roseum subsp. integrifolium* | USA: CA, Tulare Cty. | 36.43 | -118.61 | UC421147 |
| *Sedum roseum subsp. integrifolium* | USA: CA, Tulare Cty. | 36.56 | -118.51 | UC642739 |
| *Sedum roseum subsp. integrifolium* | USA: CA, Inyo Cty. | 36.56 | -118.28 | UC642744 |
| *Sedum roseum subsp. integrifolium* | USA: CA, Tulare Cty. | 36.61 | -118.71 | UC575041 |
| *Sedum roseum subsp. integrifolium* | USA: CA, Tulare Cty. | 36.64 | -118.69 | UC205186 |
| *Sedum roseum subsp. integrifolium* | USA: CA, Tulare Cty. | 36.68 | -118.41 | JEPS50736 |
| *Sedum roseum subsp. integrifolium* | USA: CA, Tulare Cty. | 36.70 | -118.40 | JEPS47999 |
| *Sedum roseum subsp. integrifolium* | USA: CA, Fresno Cty. | 36.76 | -118.39 | JEPS48001 |
| *Sedum roseum subsp. integrifolium* | USA: CA, Inyo Cty. | 36.76 | -118.36 | UC694205 |
| *Sedum roseum subsp. integrifolium* | USA: CA, Fresno Cty. | 36.80 | -118.42 | UC1564843 |
| *Sedum roseum subsp. integrifolium* | USA: CA, Fresno Cty. | 37.09 | -118.68 | UC1225456 |
| *Sedum roseum subsp. integrifolium* | USA: CA, Inyo Cty. | 37.13 | -118.50 | UC669937 |
| *Sedum roseum subsp. integrifolium* | USA: CA, Fresno Cty. | 37.15 | -118.70 | JEPS48004 |
| *Sedum roseum subsp. integrifolium* | USA: CA, Fresno Cty. | 37.15 | -118.78 | UC1215485 |
| *Sedum roseum subsp. integrifolium* | USA: CA, Inyo Cty. | 37.19 | -118.58 | CHSC27691 |
| *Sedum roseum subsp. integrifolium* | USA: CA, Fresno Cty. | 37.24 | -118.68 | UC830984 |
| *Sedum roseum subsp. integrifolium* | USA: CA, Fresno Cty. | 37.25 | -118.71 | UC1142436 |
| *Sedum roseum subsp. integrifolium* | USA: CA, Inyo Cty. | 37.27 | -118.67 | UC642605 |
| *Sedum roseum subsp. integrifolium* | USA: CA, Fresno Cty. | 37.30 | -119.18 | JEPS48005 |
| *Sedum roseum subsp. integrifolium* | USA: CA, Fresno Cty. | 37.34 | -118.90 | UC1225417 |
| *Sedum roseum subsp. integrifolium* | USA: CA, Fresno Cty. | 37.46 | -118.94 | UC1294142 |
| *Sedum roseum subsp. integrifolium* | USA: CA, Mono Cty. | 37.55 | -118.87 | CDA19330 |
| *Sedum roseum subsp. integrifolium* | USA: CA, Madera Cty. | 37.74 | -119.27 | JEPS48030 |
| *Sedum roseum subsp. integrifolium* | USA: CA, Mono Cty. | 37.81 | -119.19 | UC481452 |
| *Sedum roseum subsp. integrifolium* | USA: CA, Tuolumne Cty. | 37.89 | -119.22 | UC14462 |
| *Sedum roseum subsp. integrifolium* | USA: CA, Tuolumne Cty. | 37.90 | -119.23 | JEPS48007 |
| *Sedum roseum subsp. integrifolium* | USA: CA, Mono Cty. | 37.96 | -119.25 | JEPS111282 |
| *Sedum roseum subsp. integrifolium* | USA: CA, Tuolumne Cty. | 37.97 | -119.49 | UC1127128 |
| *Sedum roseum subsp. integrifolium* | USA: CA, Mono Cty. | 37.97 | -119.32 | UC547794 |
| *Sedum roseum subsp. integrifolium* | USA: CA, Tuolumne Cty. | 37.97 | -119.32 | UC642738 |
| *Sedum roseum subsp. integrifolium* | USA: CA, Tuolumne Cty. | 38.03 | -119.33 | UC1127129 |
| *Sedum roseum subsp. integrifolium* | USA: CA, Tuolumne Cty. | 38.09 | -119.35 | UC1127127 |
| *Sedum roseum subsp. integrifolium* | USA: CA, Mono Cty. | 38.12 | -119.33 | UC577805 |
| *Sedum roseum subsp. integrifolium* | USA: CA, Alpine Cty. | 38.40 | -119.87 | UC1127125 |
| *Sedum roseum subsp. integrifolium* | USA: CA, Alpine Cty. | 38.67 | -119.99 | UC733669 |
| *Sedum roseum subsp. integrifolium* | USA: CA, El Dorado Cty. | 38.83 | -120.04 | JEPS27609 |
| *Sedum roseum subsp. integrifolium* | USA: CA, El Dorado Cty. | 38.86 | -120.11 | UC205210 |
| *Sedum roseum subsp. integrifolium* | USA: CA, El Dorado Cty. | 38.86 | -120.08 | JEPS72357 |
| *Sedum roseum subsp. integrifolium* | USA: CA, El Dorado Cty. | 38.87 | -120.11 | JEPS48002 |
| *Sedum roseum subsp. integrifolium* | USA: CA, El Dorado Cty. | 38.89 | -120.18 | UC747400 |
| *Sedum roseum subsp. integrifolium* | USA: CA, El Dorado Cty. | 38.90 | -120.20 | UC747401 |
| *Sedum roseum subsp. integrifolium* | USA: CA, El Dorado Cty. | 38.91 | -120.10 | UC14451 |
| *Sedum roseum subsp. integrifolium* | USA: CA, El Dorado Cty. | 38.95 | -120.11 | UC571155 |
| *Sedum roseum subsp. integrifolium* | USA: CA, Placer Cty. | 39.17 | -120.23 | JEPS72358 |
| *Sedum roseum subsp. integrifolium* | USA: CA, Nevada Cty. | 39.31 | -120.31 | UC58349 |
| *Sedum roseum subsp. integrifolium* | USA: CA, Nevada Cty. | 39.32 | -120.23 | UC191153 |
| *Sedum roseum subsp. integrifolium* | USA: CA, Nevada Cty. | 39.46 | -120.55 | CHSC88574 |
| *Sedum roseum subsp. integrifolium* | USA: CA, Nevada Cty. | 39.46 | -120.55 | JEPS105982 |
| *Sedum roseum subsp. integrifolium* | USA: CA, Modoc Cty. | 41.38 | -120.23 | UC1567433 |
| *Sedum roseum subsp. integrifolium* | USA: CA, Siskiyou Cty. | 41.93 | -122.97 | JEPS81635 |
| *Sedum roseum subsp. integrifolium* | USA: CA, Siskiyou Cty. | 41.94 | -122.86 | JEPS83983 |
| *Sedum roseum subsp. integrifolium* | USA: CA, Siskiyou Cty. | 41.95 | -123.06 | JEPS82427 |
| *Sedum roseum subsp. integrifolium* | Canada: British Columbia | 49.00 | -120.25 | V21658 |
| *Sedum roseum subsp. integrifolium* | Canada: British Columbia | 49.00 | -117.00 | V12128 |
| *Sedum roseum subsp. integrifolium* | Canada: British Columbia | 49.00 | -115.00 | V100565 |
| *Sedum roseum subsp. integrifolium* | Canada: British Columbia | 49.65 | -116.17 | V90584 |
| *Sedum roseum subsp. integrifolium* | Canada: British Columbia | 50.13 | -117.42 | V12129 |
| *Sedum roseum subsp. integrifolium* | Canada: British Columbia | 52.00 | -126.00 | V119642 |
| *Sedum roseum subsp. integrifolium* | Canada: British Columbia | 52.75 | -125.25 | V146215 |
| *Sedum roseum subsp. integrifolium* | Canada: British Columbia | 54.00 | -127.00 | V95238 |
| *Sedum roseum subsp. integrifolium* | Canada: British Columbia | 54.12 | -127.50 | V151656 |
| *Sedum roseum subsp. integrifolium* | Canada: British Columbia | 54.48 | -128.45 | V86084 |
| *Sedum roseum subsp. integrifolium* | Canada: British Columbia | 55.12 | -127.58 | V97442 |
| *Sedum roseum subsp. integrifolium* | USA: AK | 56.03 | -156.70 | V171918 |
| *Sedum roseum subsp. integrifolium* | Canada: British Columbia | 56.65 | -129.62 | V168385 |
| *Sedum roseum subsp. integrifolium* | Canada: British Columbia | 57.00 | -130.00 | V145239 |
| *Sedum roseum subsp. integrifolium* | Canada: British Columbia | 59.00 | -137.00 | V93842 |
| *Sedum roseum subsp. integrifolium* | Canada: British Columbia | 59.00 | -136.00 | V141599 |
| *Sedum roseum subsp. integrifolium* | Canada: British Columbia | 59.42 | -133.75 | V159715 |
| *Sedum roseum subsp. integrifolium* | Canada: British Columbia | 59.50 | -136.50 | WTU234846 |
| *Sedum roseum subsp. integrifolium* | Canada: British Columbia | 59.80 | -136.62 | V56467 |
| *Sedum roseum subsp. integrifolium* | Canada: Yukon Territory | 60.05 | -134.70 | V200600 |
| *Sedum roseum subsp. integrifolium* | Canada: Yukon Territory | 61.00 | -138.57 | V200598 |
| *Sedum roseum subsp. integrifolium* | Canada: Yukon Territory | 61.05 | -138.52 | V100103 |
| *Sedum roseum subsp. integrifolium* | Canada: Yukon Territory | 61.58 | -138.83 | V99644 |
| *Sedum roseum subsp. integrifolium* | Canada: Northwest Territories | 62.08 | -127.58 | V90319 |
| *Sedum roseum subsp. integrifolium* | USA: AK | 62.75 | -148.75 | V106821 |
| *Sedum roseum subsp. integrifolium* | USA: AK | 63.74 | -171.69 | V44587 |
| *Sedum roseum subsp. integrifolium* | USA: AK | 63.83 | -171.75 | V38121 |
| *Sedum roseum subsp. roseum* | Canada: British Columbia | 51.97 | -131.02 | V163800 |
| *Sedum roseum var. integrifolium* | USA: WA, Okanogan Cty. | 48.99 | -119.92 | WTU332968 |
