# Supplementary figures and images for: Deep Genetic Divergence between Disjunct Refugia in the Arctic-Alpine King’s Crown, Rhodiola integrifolia (Crassulaceae)
Source: PLoS One. 2013 Nov 1;8(11):e79451. doi: 10.1371/journal.pone.0079451 (PMC3838311; doi:10.1371/journal.pone.0079451)

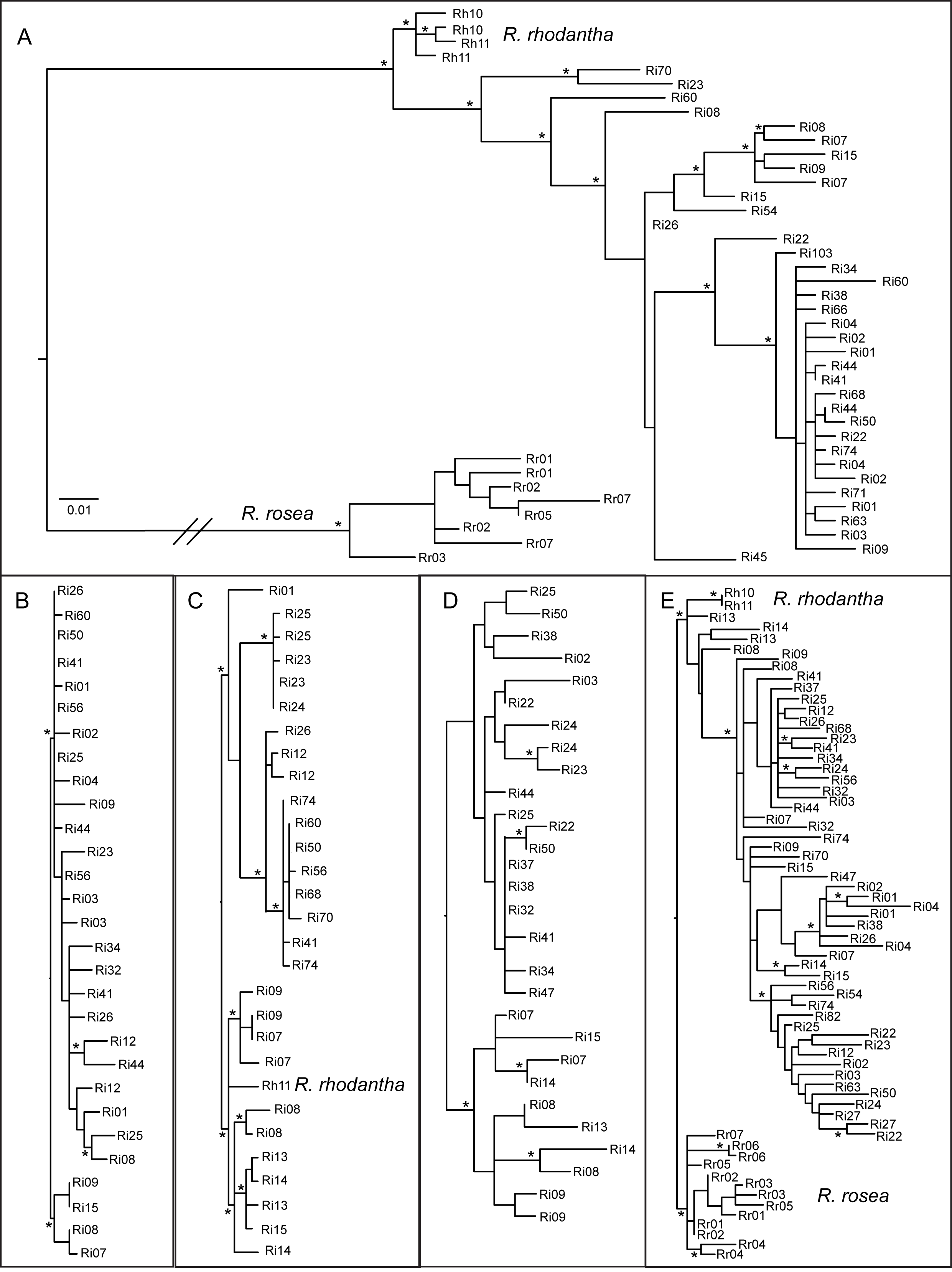

Supplement: Figure S1 — Maximum likelihood trees for each of the 5 anonymous nuclear loci. A. Rhod_1, B. Rhod_2, C. Rhod_3, D. Rhod_4, and 5. Rhod_5. Where possible, R. rosea was used as the outgroup, otherwise trees were rooted at the midpoint. All branch lengths are normalized to the scale bar showing 0.01 substitutions. The branches for R. rosea and R. rhodantha are labeled. Bootstrap values >70 are shown by an *. (TIF) [file pone.0079451.s004.tif]
